# Comparison of Transcriptomic Platforms for Analysis of Whole Blood from Ebola-Infected Cynomolgus Macaques

**DOI:** 10.1038/s41598-017-15145-7

**Published:** 2017-11-07

**Authors:** Emily Speranza, Louis A. Altamura, Kirsten Kulcsar, Sandra L. Bixler, Cynthia A. Rossi, Randal J. Schoepp, Elyse Nagle, William Aguilar, Christina E. Douglas, Korey L. Delp, Timothy D. Minogue, Gustavo Palacios, Arthur J. Goff, John H. Connor

**Affiliations:** 1 Department of Microbiology, Bioinformatics Program, National Emerging Infectious Diseases Laboratories, Boston University, Boston, MA United States; 2 Diagnostic Systems Division, United States Army Medical Research Institute of Infectious Diseases, Fort Detrick, MD United States; 3 Center for Genome Sciences, United States Army Medical Research Institute of Infectious Diseases, Fort Detrick, MD United States; 4 Molecular and Translational Sciences Division, United States Army Medical Research Institute of Infectious Diseases, Fort Detrick, MD United States; 5 Virology Division, United States Army Medical Research Institute of Infectious Diseases, Fort Detrick, MD United States

## Abstract

Ebola virus disease (EVD) is a serious illness with mortality rates of 20–90% in various outbreaks. EVD is characterized by robust virus replication and strong host inflammatory response. Analyzing host immune responses has increasingly involved multimodal approaches including transcriptomics to profile gene expression. We studied cynomolgus macaques exposed to Ebola virus Makona via different routes with the intent of comparing RNA-Seq to a NanoString nCounter codeset targeting 769 non-human primate (NHP) genes. RNA-Seq analysis of serial blood samples showed different routes led to the same overall transcriptional response seen in previously reported EBOV-exposed NHP studies. Both platforms displayed a strong correlation in gene expression patterns, including a strong induction of innate immune response genes at early times post-exposure, and neutrophil-associated genes at later time points. A 41-gene classifier was tested in both platforms for ability to cluster samples by infection status. Both NanoString and RNA-Seq could be used to predict relative abundances of circulating immune cell populations that matched traditional hematology. This demonstrates the complementarity of RNA-Seq and NanoString. Moreover, the development of an NHP-specific NanoString codeset should augment studies of filoviruses and other high containment infectious diseases without the infrastructure requirements of RNA-Seq technology.

## Introduction

Ebola virus (EBOV) is a negative sense non-segmented RNA virus of the family *Filoviridae*, genus *Ebolavirus*. EBOV is the causative agent of Ebola virus disease (EVD) in humans and non-human primates (NHPs). EVD is a very serious illness resulting in 20–90% case fatalities in humans and NHPs^1^. Disease transmission typically occurs by direct contact with bodily fluids and excreta from infected individuals. The 2014 Western Africa EBOV outbreak was the most widespread to date, suggesting mucosal routes of exposure played a large part in transmission.

NHPs, primarily cynomolgus and rhesus macaques, infected with wild-type EBOV develop a slightly accelerated, but clinically similar, presentation to human EVD, thus making them useful for studying host responses^2^. Following intramuscular infection (IM), the virus replicates to high levels in the liver and spleen. By approximately 4–5 days post-exposure, the virus is detectable at high levels in the blood and has widely disseminated to other tissues. Typically animals will succumb to disease by 6–8 days post-exposure^2^. Several recent studies have turned to focus on EBOV infection via contact with mucosal membranes or aerosol inhalation^3,4^. Indeed, modeling of different transmission routes suggests that mucosal infection is possible, but may require a greater infectious dose than is needed for IM injection^5^. Previous studies have shown 100% lethality following IM exposure to as little as 18 PFU^6^ as compared to 100 PFU needed for the oral and conjunctival routes^5^. Also, animal models have shown that the virus can cause productive infection upon entry through the lungs^7^, although the relevance of such models to natural transmission is controversial.

Transcriptomic analysis is a useful technique to study the host response to EVD. Previous work in NHPs examined the host response to infection within the circulating immune system^8–11^. Most notable was the observation of an early and robust innate immune response^10^. This is different from *in vitro* models of infection using human immortalized liver cells in which the virus is able to evade the innate immune response through interferon antagonism^12–14^. Other recent studies have used transcriptomics in whole blood samples from patients taken from the 2013–2016 EVD outbreak to better understand the progression of host responses and correlate them with survival outcomes^15,16^.

Transcriptomic data sets can also be leveraged for the development of diagnostic biomarker assays that can classify infection status and/or predict clinical outcomes, and have been demonstrated in NHPs using microarrays^9^ and miRNA^17^ expression, respectively. There has also been work to develop a biomarker for survival in human infections of EBOV through transcriptomic analysis^15^. Other work has been aimed at the development of virus-specific biomarkers for two other hemorrhagic fever viruses, Marburg virus (MARV) and Lassa fever virus (LASV), which showed distinct expression profiles at time points as early as day 1 or 3 post-exposure for LASV and MARV, respectively^18^. Host biomarker-targeted diagnostics capable of identifying EBOV infection signatures would be advantageous for patient care and outbreak control by complementing existing virus-specific nucleic acid tests and immunoassays, and potentially offering prognostic information.

In this study, we used two techniques, whole transcriptome RNA-Seq and targeted NanoString gene expression profiling, to quantify mRNA and analyze the host response to EBOV/Makona (EBOV/Mak) infection in whole blood samples obtained from cynomolgus macaques exposed by different routes of infection. We compared these platforms for mRNA quantification and demonstrated successful profiling of host gene expression, predictive classification of subjects according to infection status, and extrapolation of relative abundances of circulating immune cell populations. Although our results do not indicate that different routes of infection affect the host response to EBOV/Mak, we provide strong evidence that both RNA-Seq and NanoString yield complementary data for interrogating this response. Moreover, the significantly simplified NanoString experimental workflow and data analysis provides a valuable addition to the experimental toolkit available for monitoring correlates of infection in filovirus NHP infection models.

## Results

### NHP infection studies and sample collection

To confirm the virulence and characterize the host response in NHPs to a representative isolate of the 2013–2016 EVD outbreak, we generated a well-characterized challenge stock of virus isolated from the serum of a 2014 fatal human case in Sierra Leone (Ebola virus/H.sapiens-wt/SLE/2014/Makona-G3864.1).The sequence obtained from the clinical specimen (GenBank accession KR013754.1) was identical to 17 other passage zero sequences from the outbreak at the time of selection of the challenge stock, and eight of these came from non-survivors. In short, the sequence of SL-3864 was identical to the sequence of viruses associated with lethal disease (data not shown).

In parallel efforts to develop a model of EBOV/Mak infection that would recapitulate potential transmission modes noted during the outbreak, we performed exploratory studies in cynomolgus macaques in which we varied both exposure routes and infectivity of the inocula. Here, we report analyses on a subset of six of these animals that developed disease following exposure to virus by three different routes: intramuscular (IM), oral, and ophthalmic (Table 1). For IM exposures, animals each received 1000 or 10 PFU EBOV/Mak inoculum, respectively, by injection. By the oral and ophthalmic routes, NHPs received 100 PFU EBOV/Mak inocula dropwise. The intent of these latter exposure routes was to more realistically simulate mucosal exposure conditions using a lesser inoculum^5^. The IM exposures were included to bridge these data to existing data sets collected during various filovirus countermeasure development efforts. Following EBOV/Mak exposures, NHPs were observed for clinical manifestations of EVD as well as body temperature and weight changes. Whole blood was collected on days 0, 3, 6, and 10 post-exposure for assessment of clinical chemistry, hematology profiling, viremia determination, and also archived for downstream assays.

**Table 1:** Summary of NHP exposure routes, doses, and salient clinical findings.

NHP ID	Sex	Weight kg	Exposure Route	Inoculum PFU	Rash Day onset (severity): Localization	Lymphadenopathy Day onset (severity): Localization	Motor Dysfunction Day onset (severity)	Time to Death days
4	M	5.3	Intramuscular	10	10 (2): localized	3 (1): L man, R man, L Ax, R Ax; 10 (2): L Ax, R Ax, R Ing	11 (3)	10.74
3	M	4.7	Intramuscular	10	10 (2): localized	10 (3): L Ing	11 (1)	11.80
6	M	7.0	Intramuscular	1000	8 (2): widespread	9 (2): L Ax, R Ax, L Ing, R Ing	9 (3)	9.26
2	F	7.1	Intramuscular	1000	8 (2): limited	—	—	7.85
5	M	5.8	Opthalmic	100	10 (4): limited	10 (4): L Ing	11 (1)	12.86
1	M	5.2	Oral	100	10 (3): localized	10 (3): L Ax, R Ax, R Ing	9 (1)	9.86

Symptom severity: 1, minimal; 2, mild; 3, moderate; 4, severe. Abbreviations: PFU, plaque forming unit; L man, left mandibular; R man, right mandibular; L ax, left axial; R ax, right axial; L ing, left inguinal; R ing, right inguial.

**Figure 1:**
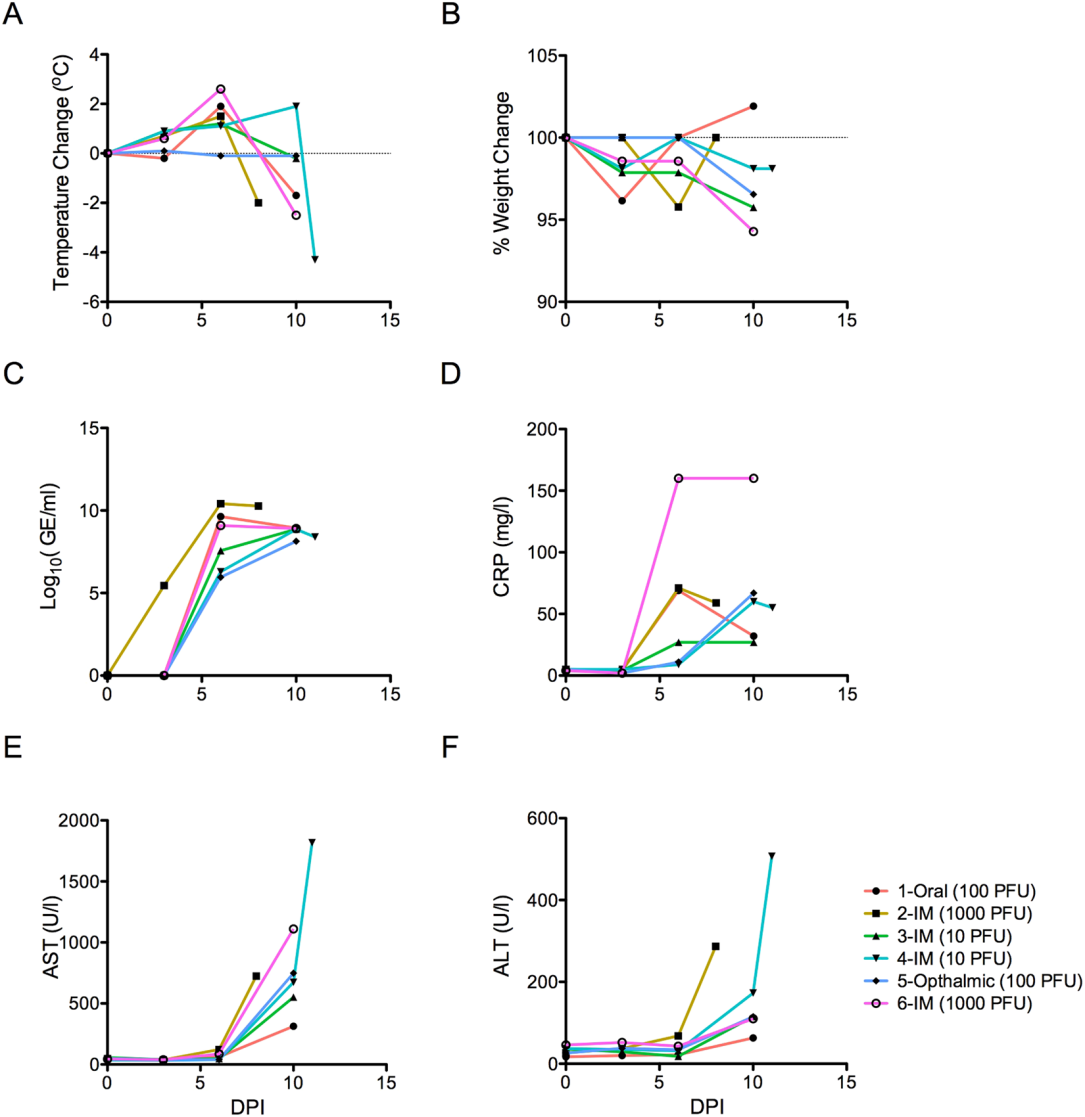
Clinical observations in NHPs exposed to EBOV/Mak. Cynomolgus macaques were exposed to EBOV/Mak according the conditions indicated in the legend. Data from each animal are plotted separately. (**A**) Changes in core body temperatures and (**B**) percent weight changes relative to pre-infection baselines for each animal are shown. (**C**) Plasma viremia expressed as log_10_ genome equivalents/ml was measured by an EBOV-specific RT-qPCR assay. Levels of (**D**) C-reactive protein, (**E**) aspartate aminotransferase, and (**F**) alanine aminotransferase in whole blood are shown for each animal.

Following EBOV/Mak exposure, the progression to EVD was consistent with previous observations in the EBOV/Kikwit (EBOV/Kik) IM exposure model (Table 1; Fig. 1). No obvious differences were observed among the exposure routes, and we acknowledge that sample numbers reported here make statistical comparisons impossible. Animals that developed symptoms of EVD had elevated temperatures and evidence of plasma viremia on day 6 post-exposure (Fig. 1A,C), showed evidence of an inflammatory response by day 6 post-exposure through elevated C-reactive protein (CRP), and elevated liver enzymes aspartate and alanine aminotransferase levels (AST, ALT; Fig. 1D–F) by day 10 post-exposure. All animals described here either succumbed to EVD or were humanely euthanized upon reaching predefined endpoints.

### Whole transcriptome sequencing analyses

**Figure 2:**
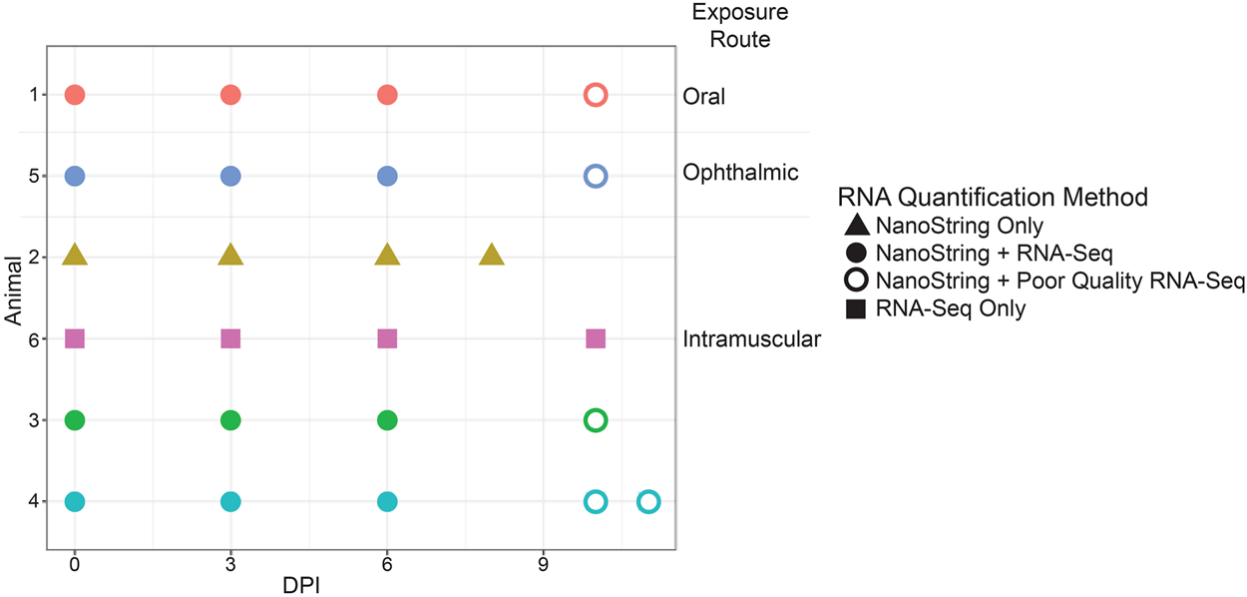
Available NHP whole blood RNA samples and gene expression analyses performed. This matrix illustrates the whole blood RNA samples analyzed by RNA-Seq and the NanoString nCounter platforms for symptomatic NHPs. The x-axis represents the day post-exposure (DPI) and each row represents a different subject. The shape and fill of the dots indicates which analyses were performed. Samples that yielded poor quality RNA-Seq were removed from analysis after quality control checks. Exposure routes are noted by labeling.

To understand the host response in circulating immune cells following NHP exposure to EBOV/Mak, total RNA isolated from previously frozen whole blood aliquots was analyzed by RNA-Seq on an Illumina HiSeq. 2500 using TruSeq library preparation technology. There was an average coverage of 23 million reads per sample used in the alignment with 90% average alignment to the Cynomolgus Macaque Genome^19^ (Supplemental Figure S1). However, poor RNA quality limited data collection from all samples of one animal (NHP 2; IM exposure), and from four of five animals on day 10 post-exposure (Fig. 2). These poor quality samples were not included in the RNA-Seq analysis.

**Figure 3:**
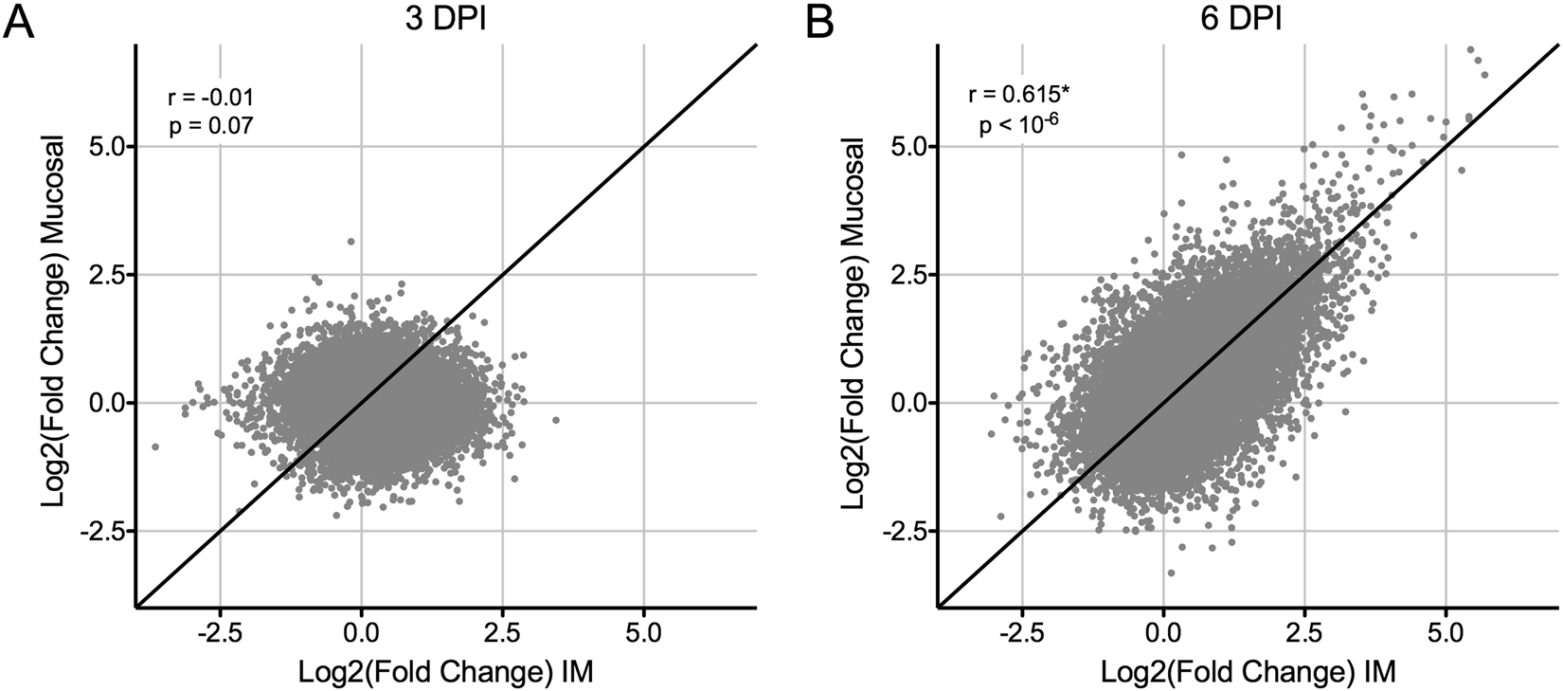
Comparison of RNA-Seq data from intramuscular and mucosal infection routes. For each gene and time point, the mean counts across all subjects in each exposure group were  used to determine the fold change in expression relative to matched pre-exposure data. Fold change data was log_2_-transformed and the mucosal and intramuscular exposures were then plotted on the x and y axes, respectively. Correlation plots for (**A**) day 3 and (**B**) day 6 post-exposure are shown with their corresponding correlation and p-value statistics. The line y = x is plotted for reference to show an ideal positive correlation. *Represents a significant correlation.

For each exposure route, IM or mucosal, we identified differentially expressed genes at each time point relative to the day 0 baselines. We then compared the various exposure routes to address whether mucosal and IM infections resulted in different gene expression profiles. By day 3 post-exposure, there was little evidence of differential gene expression in either route of infection by RNA-Seq (Fig. 3A) with a total of 11 differentially expressed genes. We next compared the transcriptional response at day 6 post-exposure between the two groups. The data showed a positive correlation between the IM and mucosal exposure routes (Fig. 3B). However, the number of differentially expressed genes that showed both a significant change between days 0 and 6, as well as a difference between IM and mucosal exposure routes, was minimal (5 genes total). Based on these data, we concluded that data from animals in all three exposure routes could be pooled for further transcriptomic analysis and method development.

### Signaling pathway changes in the NHP response to EBOV/Mak

We performed differential gene expression analysis on the RNA-Seq dataset combining gene expression across the different exposure routes on day 3, 6, and 10 post-exposure. At day 3 post-exposure, there were no genes that were significantly differentially expressed in the EBOV/Mak challenge (Supplemental Figure S2A) compared to day 0. On day 6 post-exposure, there were approximately 2000 genes significantly up-regulated (adjusted p-value < 0.05, log2(fold change) > 1) and 200 genes significantly down-regulated (adjusted p-value < 0.05, log2(fold change) < −1) (Supplemental Figure S2B). Gene set enrichment analysis (Supplemental Figure S2C) demonstrated that mRNAs with the most significant accumulation following EBOV infection were interferon stimulated genes (ISG) and cytokines. mRNAs associated with the acute phase response and apoptosis also showed significant accumulation but were not enriched until day 10 post-exposure.

The general transcriptional response of NHPs exposed to EBOV/Mak was very similar to the response seen in NHPs challenged with other EBOV variants. As an example, we performed principal component analysis on NGS datasets from this study and a study that analyzed the response of PBMCs from NHPs exposed to EBOV/Kikwit^10^. In this analysis, the first principal component (Supplemental Figure S3A), clearly separates EBOV/Kik and EBOV/Mak samples from each other. This is likely due to a number of caveats between the two datasets including blood vs. PBMC, different exposure routes and different challenge doses. Importantly, by looking at the second and third principal components (Supplemental Figure S3B), the samples begin to cluster into response groups that correlate with disease course more than they correlate with challenge virus or sample type. The first cluster (green oval) contains all the pre-exposure time points for the EBOV/Kik dataset and the pre-exposure and day 3 post-exposure points for the EBOV/Mak dataset, highlighting the similarity of pre-symptomatic animals across the different virus challenges. The second cluster represents day 4 post-exposure for EBOV/Kik and day 6 post-exposure for EBOV/Mak. This suggests that for the EBOV/Kik challenge vs. the EBOV/Mak challenge, they are most similar at day 4 post-exposure and day 6 post-exposure, respectively. This is illustrated in Supplemental Figure S3C.

In support of the symptomatic stages of disease clustering together, ISG and cytokine expression following EBOV/Mak and EBOV/Kik exposure clusters with disease severity more effectively than it clusters with the specific virus. When selected ISGs (Supplemental Figure S3D) or cytokines (Supplemental Figure S3E) were used to cluster 3 post-EBOV/Mak exposure time points (days 3, 6, and 10, where 6 and 10 are symptomatic stages) and 2 post-EBOV/Kik challenge time points (days 4 and 7 post-exposure; both symptomatic time points), the early symptomatic time points and late symptomatic time points cluster together. This shows that the gene expression changes following challenge with EBOV are primarily influenced by stage of disease and challenge variant has a minor impact.

An additional illustration of conservation of the transcriptional host response can also be seen when comparing NHP responses to the human transcriptional response^15^ to EBOV infection. Though datasets documenting the host response to EBOV in humans are much less comprehensive than those for NHPs, a comparison of the major pathways that show gene expression changes across different NHP and human datasets is shown in Supplemental Figure S2E. This comparison highlights that NHPs and humans both show a strong induction of interferon signaling and cytokines during infection. Some notable differences are in acute phase response genes where this was notably the strongest response in humans^15^ and only moderate in the NHPs. Also, in humans, there was very little evidence of neutrophil-associated transcripts whereas in both PBMC and whole blood samples from NHPs, there was a strong induction of neutrophil-associated transcripts at near terminal time points.

### Development of a targeted NanoString codeset to monitor NHP responses to infection

Although RNA-Seq sets the bar for unbiased genome-wide transcriptomics, it carries with it significant requirements for RNA quality, and for technical and bioinformatics skills to generate and analyze data. In contrast, the NanoString nCounter gene expression platform can enable profiling of up to 800 unique transcripts with a much simpler workflow, and with the ability to accommodate low quality RNA samples. Thus, we developed an nCounter codeset targeting 769 genes (Supplemental Data Table S1), primarily those involved in host immunology, inflammatory processes, and oncology (Supplemental Figure S4), and which would have maximal cross-hybridization potential with homologous transcripts among the two NHP species commonly used as filovirus animal models – cynomolgus and rhesus macaques (Supplemental Data Table S2). Out of the total gene set, twelve genes were only annotated for cynomolgus macaque, and thirteen genes were only annotated in the rhesus macaque transcriptome.

**Figure 4:**
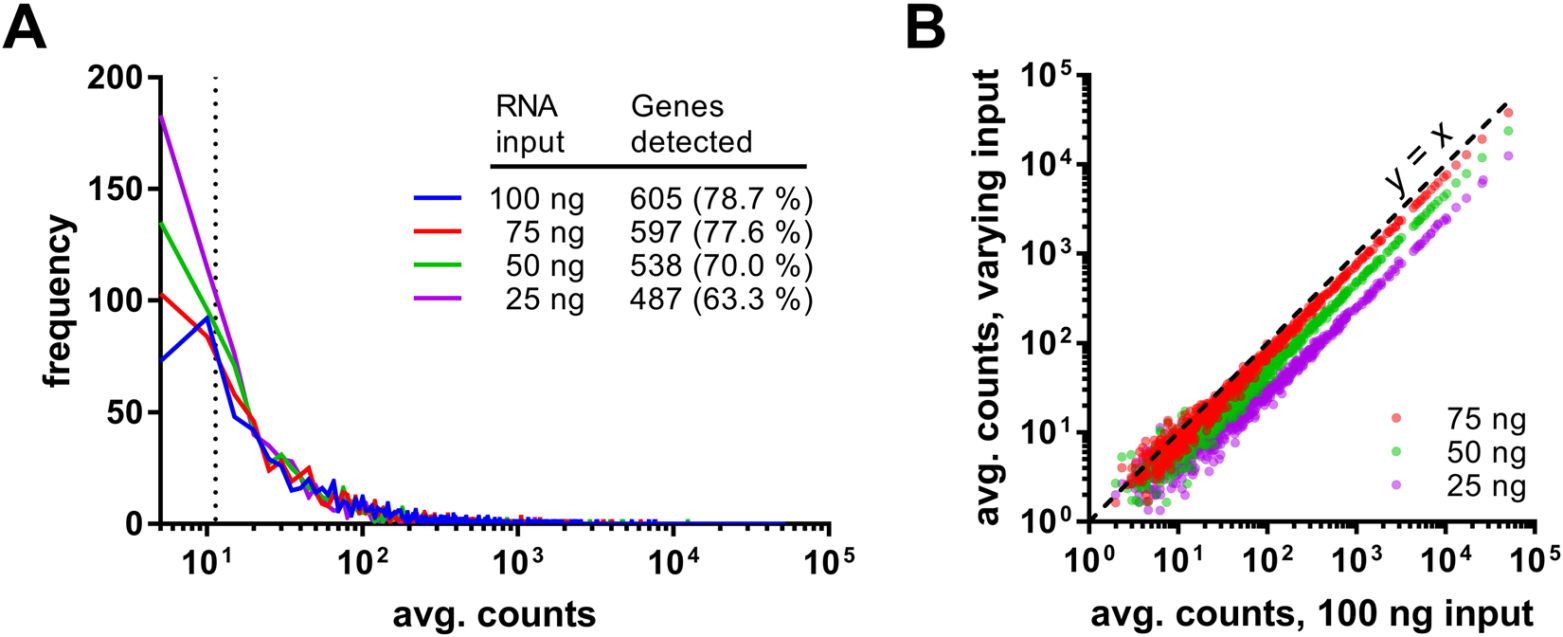
Validation of the NHP Infection Response codeset using a universal reference RNA. Varying amounts of a NHP universal reference RNA preparation derived from major organs of male and female cynomolgus and rhesus macaques were analyzed using a custom NanoString nCounter gene expression codeset targeting 769 genes. Each input RNA amount was analyzed in triplicate samples. For each RNA input amount raw data (including counts from nontargeting negative control probes) were normalized to internal positive controls and then a background threshold was defined as two standard deviations above the normalized mean of negative control probes. (**A**) Histogram of average counts per gene at varying RNA input amounts. A vertical dotted line indicates the mean of background thresholds across the four input RNA amounts. The number of genes detected and the percentage of the total 769-gene codeset is shown. (**B**) Correlation of nCounter data at varying RNA input amounts. A dashed identity line is shown (*y* = *x*).

To validate the codeset, we first analyzed varying amounts of a universal reference RNA sample representing select organs (brain, liver, pancreas, lung, and stomach) of healthy male and female cynomolgus and rhesus macaques. Following normalization to internal positive and negative controls, we were able to detect 605 genes (78.7%) with counts above the background threshold when using 100 ng of total RNA (Fig. 4A). Using as little as 25 ng RNA resulted in fewer genes being detected, but data correlation among varying amounts of input RNA was high (Fig. 4B). Our inability to detect some genes in the codeset (Supplemental Data Table S3) was not surprising given that blood cell-derived transcripts were largely absent from this reference material and this nCounter codeset specifically targeted genes involved in circulating immune responses.

**Figure 5:**
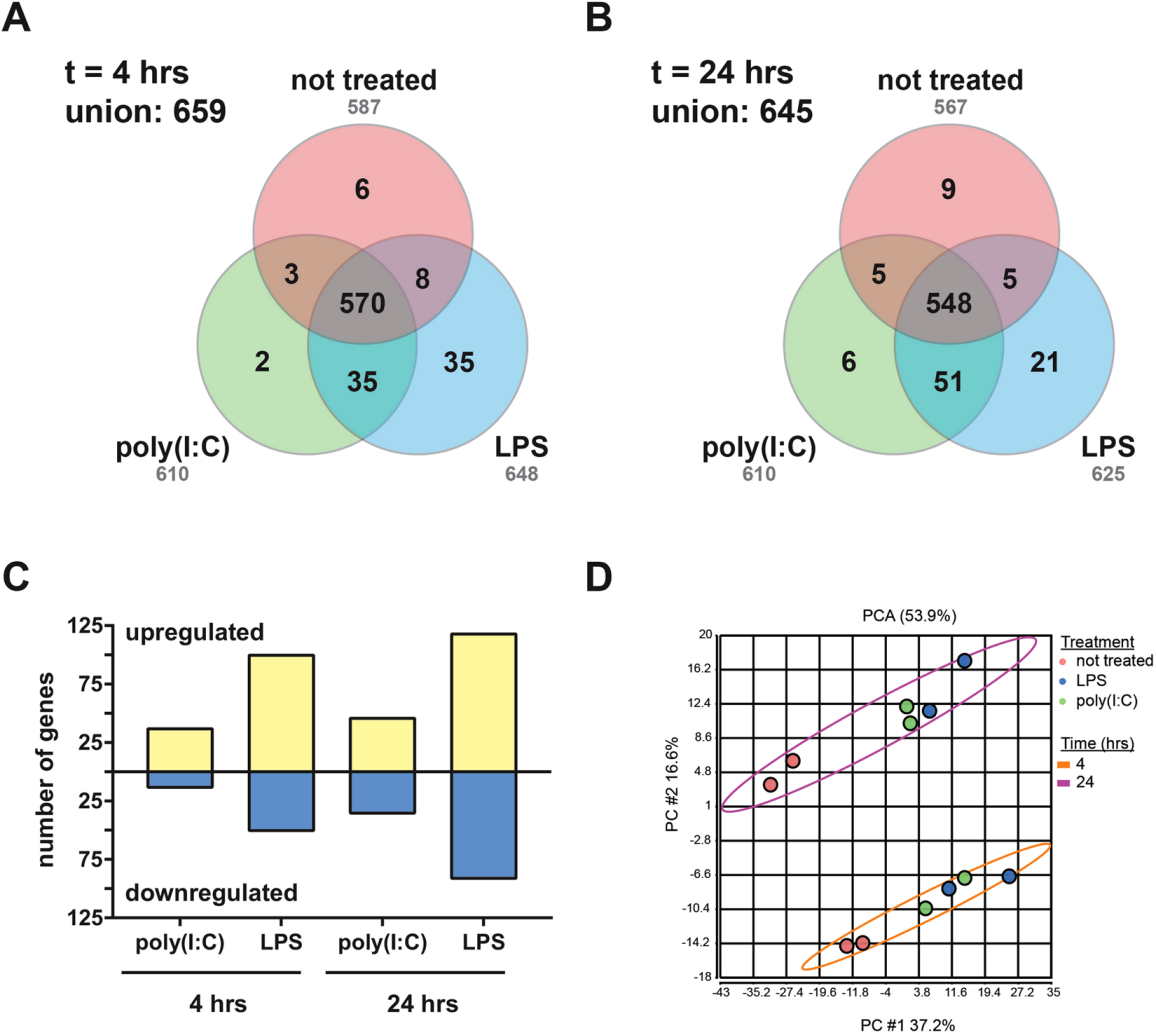
Validation of the NHP Infection Response codeset using *ex vivo* stimulated whole blood. Whole blood collected from a cynomolgus macaque stimulated with poly(I:C), LPS, or left untreated was assessed on the NanoString platform. Venn diagrams representing genes detected above background under each condition are shown for (**A**) 4 and (**B**) 24 hrs of stimulation. The total numbers of genes detected for each treatment are shown in gray text. The union of all unique detected genes under all conditions is also shown for each time point. (**C**) Differential gene expression relative to untreated samples at each time point. Normalized and log_2_-tranformed data were analyzed by ANOVA using a ± 2-fold change cutoff and an false-discovery rate adjusted p < 0.05. (**D**) Principal components analysis scatterplot of normalized data. Each treatment group is colored according to the legend. Points for each time point are bound by an ellipse.

As a second level of validation, we examined changes in cynomolgus macaque whole blood gene expression following *ex vivo* stimulation. Whole blood samples were untreated or stimulated with LPS or poly(I:C), and incubated for 4 or 24 hrs at 37 °C. Total RNA was isolated from these samples and then analyzed using our nCounter codeset. In this context, we were again able to detect the majority of genes in the panel, the union of detectable genes being 659 and 645 at the 4 and 24 hr time points, respectively (Fig. 5A,B). Across all conditions, we were able to detect 709 gene targets (92.1%) with counts above the background threshold. We also performed ANOVA to determine differential gene expression relative to untreated samples at each time point (Fig. 5C), and observed both up- and downregulation of transcripts in the treatment groups. Moreover, the total number of differentially expressed genes increased slightly between 4 and 24 hrs. Principal component analysis (Fig. 5D) demonstrated segregation of untreated and treated groups within and between each time point, indicating changes in gene expression profiles both due to treatment and time.

Finally, we compared the overlap of the previous two experiments, and found that only 24 genes were undetectable in both contexts, confirming that 745 probesets (96.9%) could detect transcripts from non-human primates (Supplemental Data Table S3). These data demonstrated the potential utility of the nCounter NHP codeset, and so we analyzed all available EBOV/Mak-infected NHP whole blood samples. In contrast to RNA-Seq, nCounter gene expression profiling generated data for all samples tested.

### Correlation of RNA-Seq and NanoString data sets

**Figure 6:**
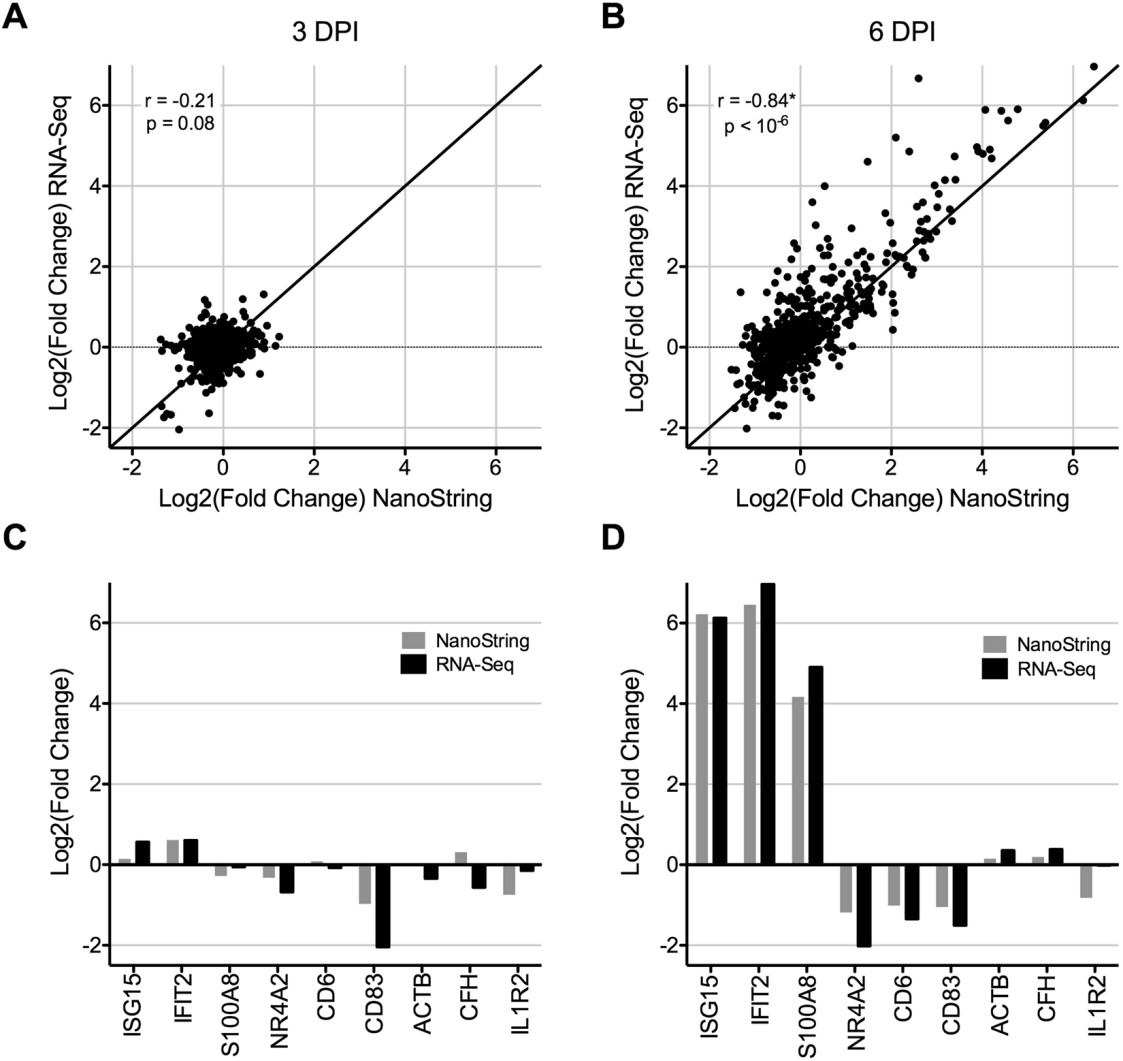
Comparison of RNA-Seq and NanoString data sets. For each gene and time point, the mean counts across all subjects measured with each platform were used to determine the fold change in expression relative to matched pre-infection data. Fold change data was log_2_-transformed and the RNA-Seq and NanoString data were then plotted on the x and y axes, respectively. Correlation plots for (**A**) day 3 and (**B**) day 6 post-exposure are shown with their corresponding correlation and p-value statistics. The line y = x is plotted for reference to show an ideal positive correlation. (**C**) Expression of representative upregulated (ISG15, IFIT2, S100A8), downregulated (NR4A2, CD6, CD83), and unchanged (ACTB, CFH, IL1R2) genes are shown for (left panel) day 3 and (right panel) day 6 post-exposure. NanoString and RNA-Seq data are shown by gray and black bars, respectively. *Represents a significant correlation.

To demonstrate that NanoString is a viable alternative to RNA-Seq in the context of EBOV/Mak infection, we determined if there was a correlation between the two data sets in the expression of the 769 genes on the NanoString panel. When examining the entire gene set averaging gene expression across the different days post-infection for each gene and gene expression platform, the correlation of count data was strongest among similar time points. As there were almost no changes in gene expression data in NanoString or RNA-Seq analysis on day 3 post-exposure, comparison of fold change relative to day 0 resulted in a poor correlation between the two platforms (Fig. 6A). However, given significant differential gene expression observed on day 6 post-exposure, there was a strong positive correlation between the two platforms (Fig. 6B). On a more granular level, there was good agreement between the two approaches among select genes that were upregulated (*e*.*g*., ISG15, IFIT2, S100A2), downregulated (*e*.*g*., NR4A2, CD6, CD83), or that remained unchanged (*e*.*g*., ACTB, CFH, IL1R2) (Fig. 6C). These genes did not show any significant expression or induction at day 3 post-exposure in either platform, but significant changes observed on day 6 were similar in both platforms.

**Figure 7:**
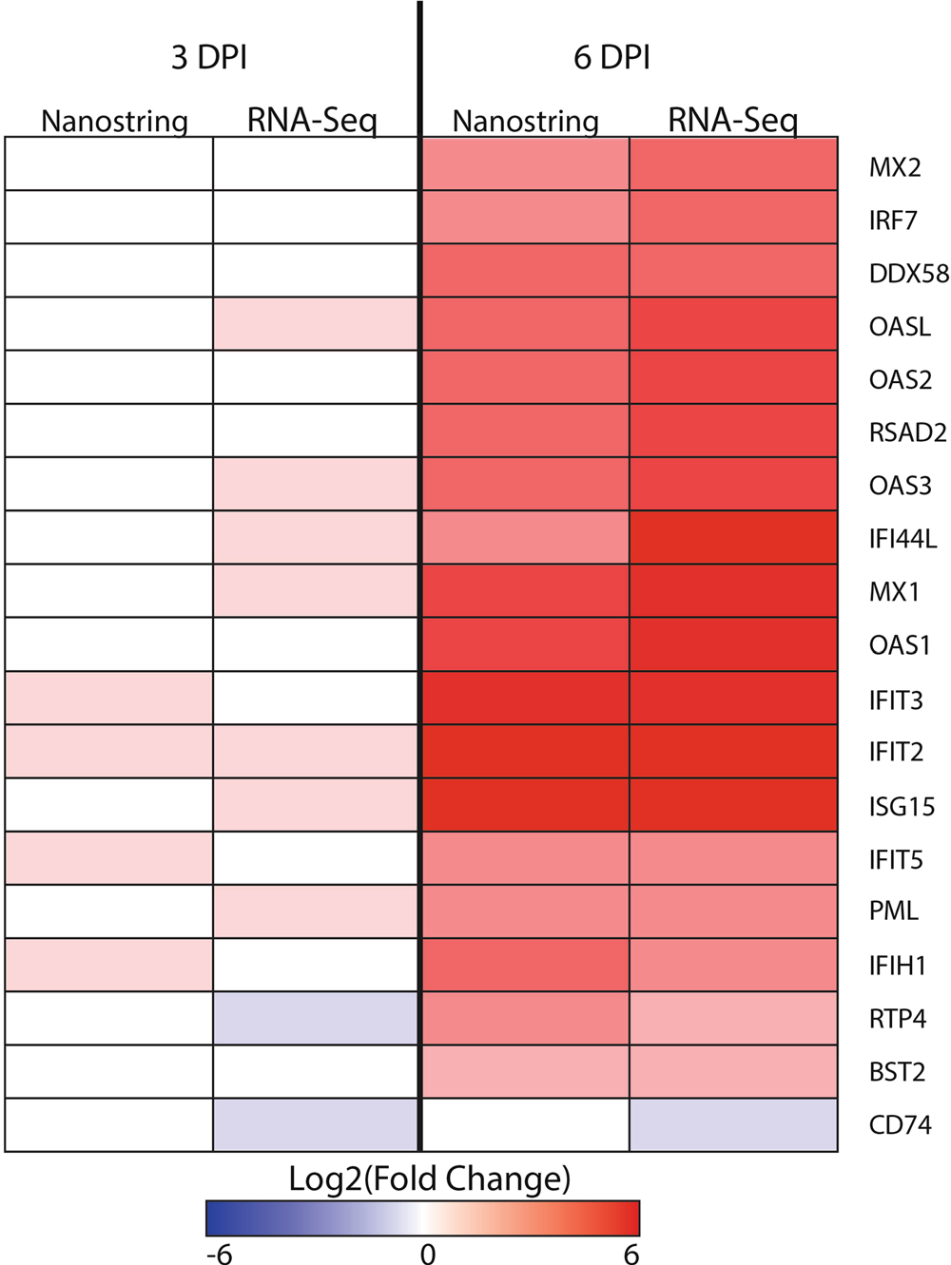
ISG response in NanoString and RNA-Seq dataset. Heatmap showing the progression of interferon-stimulated genes (ISG) in NanoString and RNA-Seq. The color represents the log 2 (fold change) with red being up-regulated and blue down-regulated. Comparative data for both day 3 (3 DPI) and day 6 (6 DPI) post-exposure are shown.

We examined the expression of a panel of interferon-stimulated genes (ISGs) in our data sets (Fig. 7). There was no significant induction of ISGs on day 3 post-exposure, but many of the classical ISGs were strongly upregulated on day 6 concurrently with the onset of viremia. Consistent with previous findings^10^, there was no detection of type I interferon mRNA in blood by RNA-Seq (Supplemental Figure S5A). However, the NanoString platform was able to detect an increase in interferon beta (IFNβ) by day 10 post-exposure in all of the animals except NHP 2, which died at day 8 post-exposure (Supplemental Figure S5B). This highlights the ability of the NanoString platform to successfully interrogate samples where RNA quality is poor, either due to severe disease or less than ideal sample handling prior to gene expression analysis.

### Verification of a PBMC-derived signature of EBOV infection in whole blood

The targeted nature of gene expression profiling on the NanoString platform is well-suited to verification of biomarkers identified by unbiased RNA-Seq or DNA microarrays. Previously, we identified 41 genes by RNA-Seq across multiple datasets that were differentially expressed and consistently up-regulated in peripheral blood mononuclear cells (PBMCs) isolated from NHPs infected with different strains of EBOV, Lassa Virus, or Marburg Virus (Supplemental Data Table S1) when compared to uninfected controls. Using both RNA-Seq and NanoString data sets, we sought to examine the ability of this gene signature to classify infection status using whole blood isolated from EBOV/Mak-infected NHPs. We focused our analysis at day 6 post-exposure, as animals were unresponsive to the infection on day 3 post-exposure both clinically and transcriptomically. This difference in disease kinetics relative to our previous data sets may be due to the lower infectious dose used here or possibly delayed disease onset for EBOV/Mak^20^.

**Figure 8:**
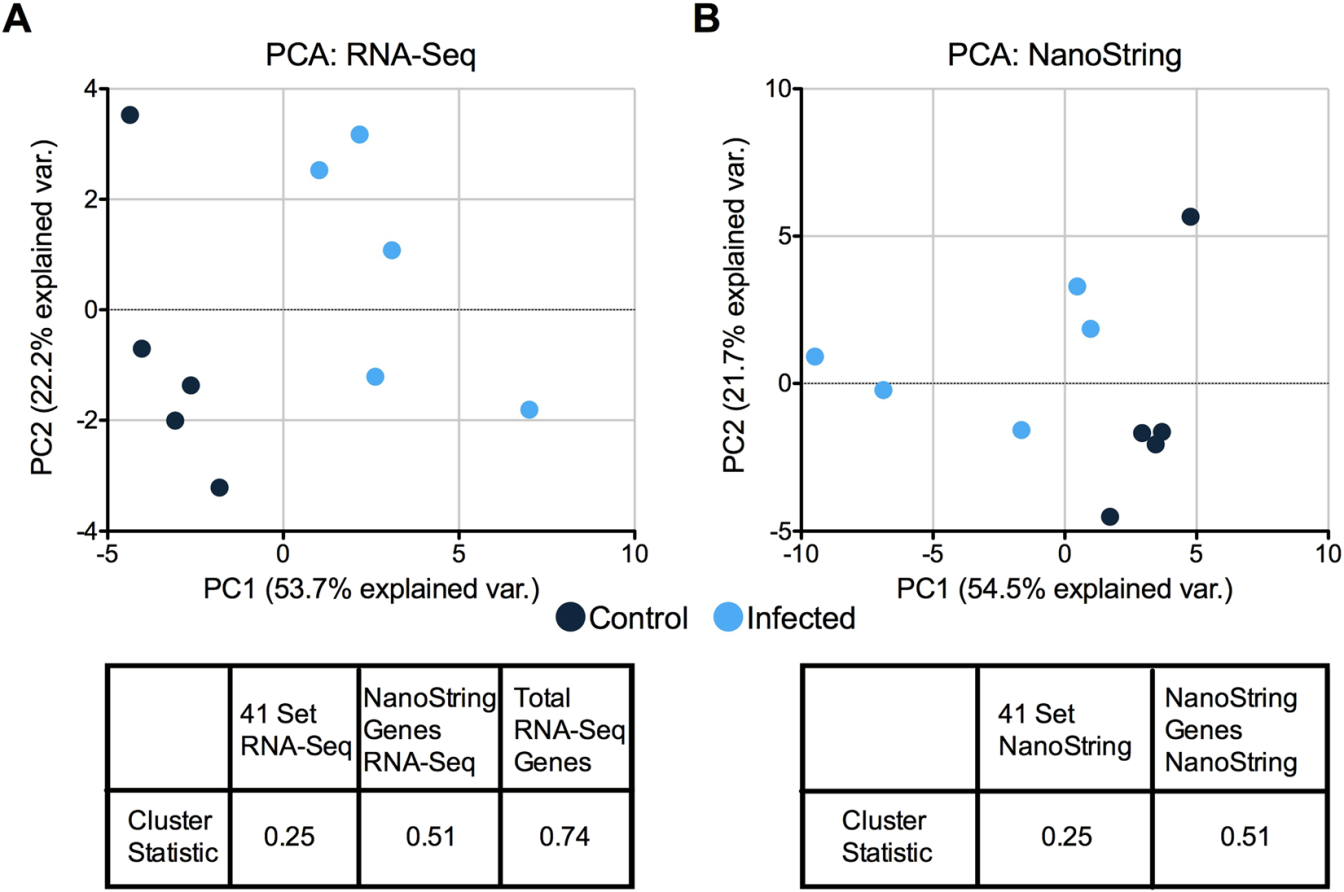
Clustering ability of the 41-gene set to distinguish infected from non-infected. Principal components analysis (PCA) plots of 41-gene EBOV infection classifier using (**A**) RNA-Seq and (**B**) NanoString expression data. Control (pre-infection baseline) and infected (day 6 post-exposure) data are highlighted in black and blue, respectively. Each table shows the clustering ability of the classifier relative to a random collection of genes present in the total NanoString or RNA-Seq data sets. The decrease from the random data was significant for both (p = 0.001 for NanoString genes and p = 0 total gene set). The decrease in the cluster statistic was again significant (p = 0) compared to 1000 random iterations.

To determine each platform’s ability to distinguish infected animals from uninfected animals using our 41-gene classifier, we examined data collected on day 6 post-exposure in comparison to pre-infection controls. We used principal component analysis to define the clusters (Fig. 8), and then calculated the ratio of the distance of each point to the center of its group cluster relative to the distance between the two clusters' centers. This was compared to 1000 iterations of randomly selecting 41 genes from the 769-gene NanoString codeset or, in the case of the RNA-Seq dataset, from the entire set of detected genes. Using data from both platforms, there was a significant improvement in the ability to cluster the two groups from each other when using the 41 gene set as opposed to a random signature (Fig. 8, Supplemental Figure S6). These data confirm the compatibility of this infection gene signature with EBOV/Mak-infected whole blood samples and gene expression data from either platform examined here. This also suggests that the 41-gene signature can be used as a corroborator of EBOV infection since it was able to cluster better than the random iterations in almost all cases (Supplemental Figure S6).

### Using Gene Expression to Predict Cell Changes

One of the benefits that the NanoString software provides is the ability to predict changes in cell types based on RNA expression to perform *in silico* cytometry analysis. Since ample hematology data was collected on infected NHPs, we were able to compare the changes predicted by *in silico* approaches to the actual data collected.

**Figure 9:**
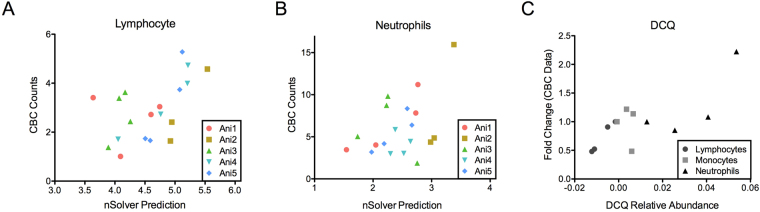
Comparison of nSolver and DCQ to the CBC data. Comparison of the nSolver prediction of changes in cell types for individual animals for (**A**) lymphocytes and (**B**) neutrophils (x-axis) compared to the CBC data for those animals (y-axis). The different animals are plotted with different colors and shapes. (**C**) A comparable plot for the DCQ data. Since DCQ using mean expression across biological replicates, the x is the DCQ relative abundance and the Y is the mean fold change from 0 DPI from the CBC data. The cell types are shown as different shapes.

We first looked at the changes predicted by the RNA-Seq data. To this end, we used Digital Cell Quantification (DCQ)^21^ which takes the fold changes of various cell marker genes as an input and predicts the changes of up to 200 different cell types using a generalized linear model. We then compared the predicted cell type changes for neutrophils, monocytes, and lymphocytes (Fig. 9C, Supplemental Figure S7). There was strong positive correlation between DCQ and hematology profiles (r = 0.86, p < 0.001). The benefit of the DCQ is that more cell types can be analyzed than any other platform.

To perform similar analysis on the NanoString data, we used the Advanced Analysis Plugin for the nSolver software. This software uses a knowledge-base specific gene expression profile from isolated human immune cells and correlates expression of many genes in isolated populations to relative cell type abundance in individual animals. A benefit of this analysis over the DCQ is that cell type changes for individual animals can be predicted and compared. Figure 9A shows the results comparing the changes in lymphocytes and Fig. 9B in neutrophils. For the lymphocytes, there was a positive correlation (r = 0.55, p = 0.01) and all animals but 1 (NHP 1) had a regression line with a slope greater than 0 (Supplemental Figure S8). The neutrophils had a less apparent correlation (r = 0.46, p = 0.04) but still had a significant correlation. In this case, two animals had regression slopes that were close to zero (NHP 3 and NHP 4). The nSolver software was able to predict individual animals changes but it correlated to the hematology data with less confidence.

## Discussion

In this study, we demonstrate the utility of a NanoString codeset targeting NHP genes to analyze the host response to EBOV/Mak infection as compared to the gold-standard of RNA-Seq. This codeset was developed to study gene expression in cynomolgus and rhesus macaques and targets 769 genes involved in host immunology and inflammatory processes. Through use of universal macaque RNA samples and *ex vivo* stimulation of macaque whole blood we were able to validate 92.1% as detectable using the novel codeset design. This codeset was used to profile the circulating immune response to EBOV infection in cynomolgus macaques and was compared to traditional RNA-Seq analysis to determine usability for differential gene expression analysis, circulating cell type predictions, and functionality as an infection classifier. The NanoString platform affords the opportunity to perform highly multiplexed and targeted gene expression with a relatively lower resource investment compared to RNA-Seq. Moreover, assays can be performed using crude RNA samples and do not require any library preparation, significantly decreasing the technical burden. Our experience also suggests that NanoString assays are robust under conditions of low RNA quality, suggesting that this approach may allow analysis of samples collected in less than ideal conditions. Notably, we have successfully implemented the NanoString workflow in a BSL-3 laboratory, and this could conceivably be accomplished in BSL-4 as well, thereby enabling transcriptomic studies of Select Agents such as EBOV without having to develop validated sample inactivation protocols.

Our data highlighted that transcriptomic analyses of whole blood and purified immune cell subsets resulted in similar gene expression profiles in EBOV-infected NHPs. Previous studies have described global gene expression of peripheral blood mononuclear cells (PBMCs) from EBOV-infected NHPs^8,10,22^ and in humans^15,16^. When comparing these admittedly disparate PBMC and whole blood transcriptomes, similar immune responses were identified (Supplemental Figures S2E and S3). The similarity of host responses to EBOV infection includes a strong induction of ISGs and strong up-regulation of cytokines, suggesting that the NHP challenge model accurately reflects many aspects of the human response to infection and that the different EBOV variants do not evoke unique host responses.

There were important differences notable between the human and NHP responses. We did not observe a strong induction of the acute phase response in EBOV/Mak challenged animals, even at the very endpoint of disease. Another notable difference is the strong evidence of neutrophil increases in both the CBC and computational predictions in the RNA-Seq and NanoString in the NHPs. In the human infections, there was no strong induction of neutrophils noted in either survivors or fatal cases of infection.

In this study, the use of two transcriptomic platforms provided cross-validation and helped to provide strong evidence that NanoString analysis can be used to provide a broad analysis of the host response. The benefit of using RNA-Seq is the acquisition of a fairly unbiased view of the RNA present and detection of upwards of 20,000 transcripts. However, RNA-Seq is dependent on high RNA quality and can have a low signal:noise ratio making lowly expressed transcripts difficult to detect.

In contrast, our NanoString probeset showed good detection of RNA even in highly degraded RNA. One example of this is the detection of the interferon beta transcript (Supplemental Figure S3) at day 10 post-exposure in NanoString where RNA-Seq could not be performed or had low quality results. The ability to perform gene expression assays on degraded samples is especially important when considering samples taken from late stages of hemorrhagic disease or in an outbreak setting. Proper care and handling of samples is difficult and samples often have to be transported before processing can begin, resulting in low quality^23^. Our results suggest that NanoString assays like the one developed here can allow mRNA quantification from clinical samples collected in low-resource environments.

We also used this dataset to test whether we can classify animals by infection status solely based on the host response. Similar work has been used to identify host gene expression biomarkers to diagnose viral or bacterial acute respiratory illness in clinical cases without detection of the causative agent^24^. There has also been research done showing that without the need to detect the causative agent, it is possible to identify different hemorrhagic fever viruses from each other^18^. Included in the NanoString set was a set of 41 genes identified through analysis of PBMC datasets from Lassa, Marburg, and Ebola virus-infected animals that we investigated as markers of progressive EBOV infection. We analyzed these genes using both RNA-Seq and NanoString and found that both platforms showed strong clustering of infected animals compared to uninfected controls. This clustering was stronger than randomly selecting genes suggesting that these genes are an optimal set of genes to detect infection (Fig. 8). This provides evidence of a biomarker signature that will identify symptomatic animals through analysis of the host response and without the need to detect the causative agent.

Genes that are present in the viral respiratory infection^24^ and Lassa/Marburg classifiers^18^ contain many interferon-stimulated genes similar to the EBOV classifier presented here signifying that the interferon response is a strong early indicator of viral infection. However, the interferon response is not specific to EBOV infection and is a general response to any viral infection. To address this, included in the probe set are 8 genes that should have a unique pattern of expression in EBOV/Kikwit when compared to Marburg and Lassa based on comparison of expression to previous studies^9,10,18^. To determine if these genes are truly unique to EBOV infection, further testing would need to be carried out comparing the response in EBOV infection compared to Marburg, Lassa, and other viral infections. Also, within this analysis the classifiers were not able to predict infection until after the onset of viremia. In previous datasets, there is evidence of gene expression changes present before the onset of viremia^10,11,25^. In this dataset, there was no evidence of any differential expression by day 3 post-exposure. Further testing would need to be performed on sampling days between day 3 and day 6 post-exposure to determine if the signature still is present before the onset of viremia.

The ability to extend analysis beyond gene expression is especially valuable when limited sample is available for multiple assays (*e*.*g*., RT-qPCR, blood chemistry, hematology, serology). Both the nSolver Advanced Analysis Plugin (NanoString) and DCQ^21^ (RNA-Seq) cell prediction algorithms were able characterize relative changes in cell type abundances using mRNA expression levels. The nSolver system was able to predict changes on an individual animal basis whereas the DCQ data was able to predict changes over all the animals combined. The nSolver system has only a few cell types that can be analyzed and is limited based on its prior information matrix (although this can be modified with additional data). The DCQ approach utilizes the entire Immgen knowledge base to look for over 200 different cell types and can be easily manipulated to use a human background of expression data from isolated cell types. Both platforms correlate well with traditional hematology data, which was consistent with previously reported changes during EBOV infection. Neutrophilia is a well characterized component of end stage EVD and lymphopenia is often observed^1^. Both of these were detected in the hematology data as well as by DCQ and the nSolver software. This also suggests that similar to other EBOV infections, infection with EBOV/Mak causes similar changes in circulating immune cells.

The current study is a novel analysis of whole blood transcriptomes of NHPs infected with EBOV/Mak. The study was limited, as samples were not taken when the animals had a strong host response but were still pre-viremic. There have been many studies showing that early gene expression of many innate immune genes occurs before the onset of viremia^9,10^. Future studies with earlier and more frequent sample collection would help to confirm similar kinetics of the host response in the context of EBOV/Mak infection. Additionally, as the number of NHPs that had productive infection by individual mucosal routes was limited, future studies would benefit from large cohorts in order to potentially compare and contrast the host responses in these exposures.

## Methods

### Biocontainment

All activities using infectious EBOV specimens and cell culture materials, as well as housing and manipulations of EBOV-exposed NHPs, were performed in BSL-4/ABSL-4 registered laboratory suites at USAMRIID.

### Virus stock

A working seed bank of the isolate Ebola virus/H.sapiens-wt/SLE/2014/Makona-G3864.1 (EBOV/Mak) was produced from a master stock that was made from an isolate from serum of a 2014 Sierra Leone fatal human case (SL 3864.1). The working stock was produced by infecting Vero E6 cells at a multiplicity of infection of 0.001 in T175 flasks. Virus was diluted and propagated in Eagle’s Minimum Essential Medium (EMEM) supplemented with 2% [vol/vol] heat-inactivated fetal bovine serum (∆FBS; HyClone), 2 mM L-glutamine (HyClone), 100 IU penicillin (Cellgro), 1000 µg/ml streptomycin (Cellgro), and 1.25 µg/ml amphotericin B (ThermoFisher Scientific). On day 9 post-inoculation, the media was harvested, an additional 8% ∆FBS added after clarified by centrifugation, and aliquoted into 1-ml single-use cryovials. The preparation was a Vero E6 passage 2 stock and contained an average of 4.02 × 10^6^ plaque-forming units per ml (PFU/ml) of infectious virus. This stock was evaluated for sterility on trypticase soy broth and chocolate agar plates and tested for mycoplasma and endotoxin levels using Lonza’s Endpoint Chromogenic Limulus Amebocyte Lysate (LAL) test and MycoAlert biochemical assay. The stock was determined to have no detectable mycoplasma, endotoxin or adventitious agents based on the assays and techniques used. Total RNA isolated from this stock was sequenced and tested in a number of real-time reverse-transcription polymerase chain reaction (RT-qPCR) assays to include those specific for each filovirus. No known contaminates were detected when sequencing the stock, and identity was confirmed by RT-qPCR. By electron microscopy, virions exhibited morphological characteristics typical of filoviruses in that they appeared as either long filamentous or rod forms, sewing needle-shaped, or in shorter mace-shaped configurations. Particles varied in length with a uniform diameter of approximately 80 nm.

### Ethics statement

Research was conducted under an IACUC-approved protocol in compliance with the Animal Welfare Act, PHS Policy, and other Federal statutes and regulations relating to animals and experiments involving animals. The study was also sent to ACURO for review and approval. The facility where this research was conducted is accredited by the Association for Assessment and Accreditation of Laboratory Animal Care, International and adheres to principles stated in the Guide for the Care and Use of Laboratory Animals, National Research Council, 2011. Humane endpoints were used during all studies, and NHPs were humanely euthanized when moribund, according to predefined clinical endpoints using a scoring system.

### Study animals, routes of exposure, and periodic monitoring

Six Chinese-origin cynomolgus macaques (*Macaca fascicularis*) were used for this study (age range: 3–9 years). Prior to initiation of the study, the animals were found to be negative for herpes B virus, simian retrovirus types-1, −2, and −3, simian immunodeficiency virus, and simian T-cell leukemia virus type-1 and negative for EBOV-reactive antibodies.

Two animals each were challenged with 1000 or 10 PFU of EBOV/Mak via intramuscular inoculation. Two animals were each challenged with 100 PFU via the ophthalmic and oral routes: For the oral exposures, 500 µl of liquid inoculum was placed in the mouth. For the ophthalmic exposures, 250 µl of inoculum was placed in each eye. All viruses were diluted in media to achieve the desired concentrations. The animals were observed daily and evaluated for clinical signs of disease. Additionally, physical examinations and phlebotomy were performed longitudinally throughout the course of the study. Blood samples were collected at various time points throughout the study for analysis of chemistry and hematological parameters, detection of viral genomes by RT-qPCR, and assessment of antibody responses.

### Hematology

Whole blood collected in K_3_-EDTA tubes was obtained for assessment of various hematological parameters. Total white blood cell (WBC) counts, WBC differentials, red blood cell counts, platelet counts, hematocrit values, total hemoglobin, mean cell volume, mean corpuscular volume, and mean corpuscular hemoglobin concentration were determined using an ADVIA 120 Hematology System (Siemens).

### Chemistry

Serum was isolated from whole blood samples and used to assess concentrations of alanine aminotransferase (ALT), aspartate aminotransferase (AST), alkaline phosphatase (ALKP), gamma-glutamyltransferase (GGT), blood urea nitrogen (BUN), and creatinine (CRE) using a Vitros 350 chemistry system (Ortho Clinical Diagnostics).

### Inoculum quantification

The dose of virus delivered to each group of animals was quantified by plaque assay. Vero E6 cells were plated in six-well cluster plates at 85–100% confluency prior to use. Virus controls were thawed at room temperature. Both viral controls and inocula prepared for animal challenges were serially diluted from 10^−1^ to 10^−6^ using Minimal Essential Media (MEM) supplemented with 10% [vol/vol] ∆FBS, 100 IU penicillin (Corning), 1000 µg/ml streptomycin (Corning). Virus positive controls and the 1000 PFU dose were diluted from 10^−1^ to 10^−6^, while the 10 PFU doses were diluted from 10^0^ to 10^−5^. Each dilution was plated in triplicate wells. The plates were incubated at 37 °C with 5% CO_2_ with gentle rocking every 15 min for a total of 60 min. A primary overlay consisting of 0.5% [wt/vol] agarose (Seakem ME, Lonza), Eagle’s Basal Medium with Earle’s Salts, 10% [vol/vol] ∆FBS, and 100 IU penicillin (Corning), 1000 µg/ml streptomycin (Corning) (collectively, EBME Complete) was prepared and 2 ml was added to each well. The plates were incubated at 37 °C with 5% CO_2_ for 7 days. Next, 2 ml of a secondary overlay consisting of 0.5% [wt/vol] agarose, EBME Complete, and 4% neutral red (Gibco) was added to each well and incubated at 37 °C with 5% CO_2_. Plaques were manually enumerated on day 9 using a light box.

### Viral load analysis by RT-qPCR

Freshly collected plasma samples (100 µl) were combined with 300 µl TRIzol LS (ThermoFisher Scientific). These samples (i.e., the vials) were surface decontaminated with 5% [vol/vol] solution of MICRO-CHEM PLUS (National Chemical Laboratories) and removed from BSL-4 to BSL-2 containment in accordance with USAMRIID standard operating procedures. RNA was subsequently extracted using the QIAamp Viral RNA Mini Kit (Qiagen). Viral genome equivalents per ml (GE/ml) were quantified using an EBOV-specific RT-qPCR assay and a synthetic RNA standard curve as previously described^26^.

### RNA isolation from whole blood

Previously flash-frozen whole blood samples were thawed, and then two matched 250-µl aliquots were each combined with 750 µl TRIzol LS, and removed from BSL-4 containment as described previously. For RNA-Seq analyses, RNA was isolated from whole blood in TRIzol LS using the PureLink RNA Mini Kit (ThermoFisher Scientific) and RNA was evaluated for quality on the Agilent 2200 TapeStation. For NanoString analyses, total RNA was isolated using the miRNeasy Mini Kit (Qiagen) and then quantified using the Qubit RNA HS Assay Kit (ThermoFisher Scientific).

### RNA-Seq

Libraries were generated on the Sciclone G3 Liquid Handling Robot (PerkinElmer) using the TruSeq Stranded Total RNA Library Prep Kit (Illumina). Library quality was evaluated on the Agilent 2200 TapeStation 2200 and quantified by qPCR using the KAPA Complete (Universal) qPCR kit (Kapa Biosystems) for Illumina libraries. Libraries were diluted to 12 pM and cluster generation was performed on the Illumina cBot. Libraries were sequenced on the HiSeq. 2500 using the paired end 2 × 100 bp, dual-index format.

### NanoString targeted gene expression

Total RNA samples were analyzed using a custom-designed NanoString nCounter gene expression codeset targeting 769 NHP genes. For each gene, a single probeset was designed with predicted cross-hybridization to homologous genes for cynomolgus macaque and rhesus macaque (*Macaca mulatta)*. Probeset-target RNA hybridization reactions were performed according to the manufacturer’s protocol. For each hybridization reaction, 100 ng total RNA was used or any quantity that was present in a 5-µl aliquot of purified RNA if less than 100 ng (mean: 92.5 ng, range: 32.5–100 ng). Purified probeset-target RNA complexes from each reaction were processed and immobilized on nCounter Cartridges using an nCounter *MAX* Prep Station and transcripts were quantified on the Digital Analyzer (GEN 2).

### NanoString codeset validation

For initial testing, a reference sample consisting of RNA isolated from lysates of major organs (minimally brain, liver, pancreas, lung, and stomach) from adult male and female cynomolgus and rhesus macaques was used (Primate Universal RNA Tissue Lysate, Adult Normal; Novus Biologicals). Varying amounts of RNA (100, 75, 50 and 25 ng per hybridization reaction) were analyzed in triplicate using the NanoString nCounter NHP gene expression codeset as described.

To examine the performance of the NanoString codeset under simulated infection conditions, whole blood from a single cynomolgus macaque was stimulated *ex-vivo* with lipopolysaccharide (LPS) or polyinosinic:polycyticylic acid (poly I:C) to induce changes in gene expression. Whole blood collected in K_3_-EDTA tubes was diluted 1:5 with RPMI 1640 with L-glutamine (Cellgro). For stimulations, LPS or poly I:C was added to a final concentration of 1.0 or 5.0 µg/ml, respectively. Samples were added to 12-well cluster plates and incubated at 37 °C with 5% CO_2_. Untreated blood samples were included as controls. Samples were collected at 4 and 24 hrs after stimulation by gently pipetting up and down to resuspend adherent cells and transferred to a microcentrifuge tube. Following centrifugation at 1200 × *g* for 10 min, the supernatants were removed, and the pellets were resuspended in 0.5 ml H_2_O. For each sample, 1.5 ml TRIzol LS was used to lyse any adherent cells in its corresponding well and then combined with the resuspended pellet. Samples were stored at −80 °C until total RNA was isolated using the miRNeasy Mini Kit (Qiagen) and then quantified using the Qubit RNA HS Assay Kit (ThermoFisher Scientific). Total RNA samples were analyzed using the NanoString nCounter NHP gene expression codeset. For each hybridization reaction, 100 ng total RNA was used, or any quantity that was present in a 5-µl aliquot of purified RNA if less than 100 ng. This experiment was repeated twice using blood from the same macaque collected approximately six weeks apart.

### RNA-Seq data processing

The whole blood data was processed with the following. The raw FASTQ files were trimmed to remove low quality bases using the FASTX-Toolkit version 0.0.14^27^. Parameters used were –l 40, -q 33 (trimming), -q 20 (filtering), -p 80. Reads were then aligned to the *Macaca fascicularis* genome version 5.0^19^ using Bowtie 2 version 2.2.6^28^ and TopHat version 2.1.0^29^ using the default parameters for alignment. Finally, count tables were generated using HTSeq version 0.5.4^30^ with –stranded set to no. The PBMC data of EBOV/Kik was processed according to^10^.

Count tables were read into R version 3.2.2^31^. Genes that had fewer than 10 counts across all samples were removed to prevent zero count rows for skewing the normalization. Data was normalized using the DESeq. 2^32^ rlog normalization. After initial normalization, samples were removed if their median value was greater than 2 standard deviations of the mean of the median normalized counts. The data was then normalized again. This was repeated until the standard deviation of the median normalized counts across samples was less than 10% of the mean. This allowed for the removal of samples that showed evidence of poor quality sequencing.

### NanoString data processing

Data for the *ex vivo* stimulation study and the EBOV/Mak infection studies were processed and analyzed independently as follows: nCounter .RCC files for each sample were imported into nSolver 2.5 for review of quality control metrics (all passed). The spike-in positive control geometric mean normalization factor (without negative control background subtraction) was determined using nSolver for each sample. Using GraphPad Prism 7, the global background of raw negative control probe counts was determined across all samples (mean + 2 standard deviations). A normalized global background threshold was then calculated by first multiplying each sample’s positive control geometric mean normalization factor by the raw global background threshold, and then calculating the mean + 2 standard deviations.

To identify stable reference genes to normalize data for varying RNA input, we used NormFinder^33^. Positive control geometric mean normalized data for all genes was exported to Microsoft Excel, and then filtered to include only genes where all samples had counts above the normalized global background threshold. This was done to remove data bias by undetected genes in some samples. This filtered data set (136 genes), was analyzed with the NormFinder Excel add-in and the top five most stable genes were selected in each study. Raw counts from these genes, along with the spike-in positive controls, were then used to perform both positive control and reference gene normalization of the raw data in nSolver. These data were then exported in.CSV format for gene expression analyses.

### Bioinformatics analysis

Count data was converted into counts per million mapped reads (CPM) through the fpm function in DESeq. 2. A pseudo count of 1 was added to all counts (both NanoString and RNA-Seq CPM) before the log2 value was calculated and the log2 fold change calculated. Differential expression analysis was done for the RNA-Seq data using the DESeq. 2 negative bionomial model to generate a Benjamini-Hochberg corrected p-value for significantly differentially expressed genes. Correlation values were calculated using the cor() function in R and p-values generated using the cor.test() function.

A 41 gene biomarker set was determined previously as genes identified to be significantly up-regulated in multiple EBOV-infected macaque PBMC transcriptomic datasets. Some of the genes were identified as common to other hemorrhagic fever viruses such as Lassa and Marburg. These genes were found to be highly reproducible across multiple datasets as up-regulated before the onset of viremia. To determine the clustering ability of the 41 gene biomarker set, a cluster statistic was calculated using the points positioning from a principal component analysis using the built-in R function. After calculating the principal components that contributed to 95% of the explained variance when centered and scaled, a mean center point for each cluster was calculated. Next, the squared mean Euclidian distance of each point to its center was calculated to determine how far points were from the center. Then, the squared distance of the centers was calculated. The final statistic was the ratio of the squared mean distance to the center over the squared distance between the centers. For the simulations, 41 genes were chosen at random to calculate the statistic. Significance was determined by the percentage of random simulations that fell below the biomarker statistic with a cutoff of 5%.

To extrapolate cell type changes from the RNA-Seq data, digital cell quantification package ComICS^34^ in R was used. An input of log2 fold change compared to 0 DPI was used as an input, default parameters used, and a split ratio of 0.5. For the NanoString data, the nCounter Advanced Analysis software was used to determine cell type changes for each animal.

### Data availability

Raw sequencing files are available through NCBI under GEO GSE99463. Significant differentially expressed genes can be found in Supplemental Table S4. NanoString normalized count data can be found in Supplemental Table S5.

## Electronic supplementary material

Supplemental Figures

Supplemental Data Table 1

Supplemental Data Table 2

Supplemental Data Table 3

Supplemental Data Table 4

Supplemental Data Table 5
