# Supplementary material for: Comparison of Transcriptomic Platforms for Analysis of Whole Blood from Ebola-Infected Cynomolgus Macaques
Source: Sci Rep. 2017 Nov 7;7:14756. doi: 10.1038/s41598-017-15145-7 (PMC5676990; doi:10.1038/s41598-017-15145-7)
Supplement: Supplementary file 1 — Supplemental Figures [file 41598_2017_15145_MOESM1_ESM.pdf]

Supplemental Data

Comparison of Transcriptomic Platforms for Analysis of Whole Blood from Ebola-Infected Cynomolgus Macaques

Emily Speranza[1#], Louis A Altamura[2#], Kirsten Kulcsar[3], Sandra L Bixler[4], Cynthia A Rossi[2], Randal J Schoepp[2], Elyse Nagle[3], William Aguilar[2], Christina E Douglas[2], Korey L Delp[2], Timothy D Minogue[2], Gustavo Palacios[3], Arthur J Goff[5]*, John H Connor[1]*

[1]Department of Microbiology, Bioinformatics Program, Boston University, Boston MA
[2]Diagnostic Systems Division, United States Army Medical Research Institute of Infectious Diseases, Fort Detrick, MD
[3]Center for Genome Sciences, United States Army Medical Research Institute of Infectious Diseases, Fort Detrick, MD
[4]Molecular and Translational Sciences Division, United States Army Medical Research Institute of Infectious Diseases, Fort Detrick, MD
[5]Virology Division, United States Army Medical Research Institute of Infectious Diseases, Fort Detrick, MD

* Corresponding Authors
[#]Contributed Equally

Emily Speranza: speranza@bu.edu
Louis A Altamura: louis.a.altamura2.ctr@mail.mil
Kirsten Kulcsar: kirsten.kulcsar@gmail.com
Sandra L Bixler: sandra.l.bixler.ctr@mail.mil
Cynthia A Rossi: cynthia.a.rossi.civ@mail.mil
Randal J Schoepp: randal.j.schoepp.civ@mail.mil
Elyse Nagle: elyse.r.nagle.ctr@mail.mil
William Aguilar: william.aguilar@nih.gov
Christian E Douglas: christina.e.burrows.ctr@mail.mil
Korey L Delp: korey.l.delp.ctr@mail.mil
Timothy D Minogue: timothy.d.minogue.civ@mail.mil
Gustavo Palacios: gustavo.f.palacios.ctr@mail.mil
*Arthur J Goff: arthur.j.goff.civ@mail.mil
*John H Connor: jhconnor@bu.edu

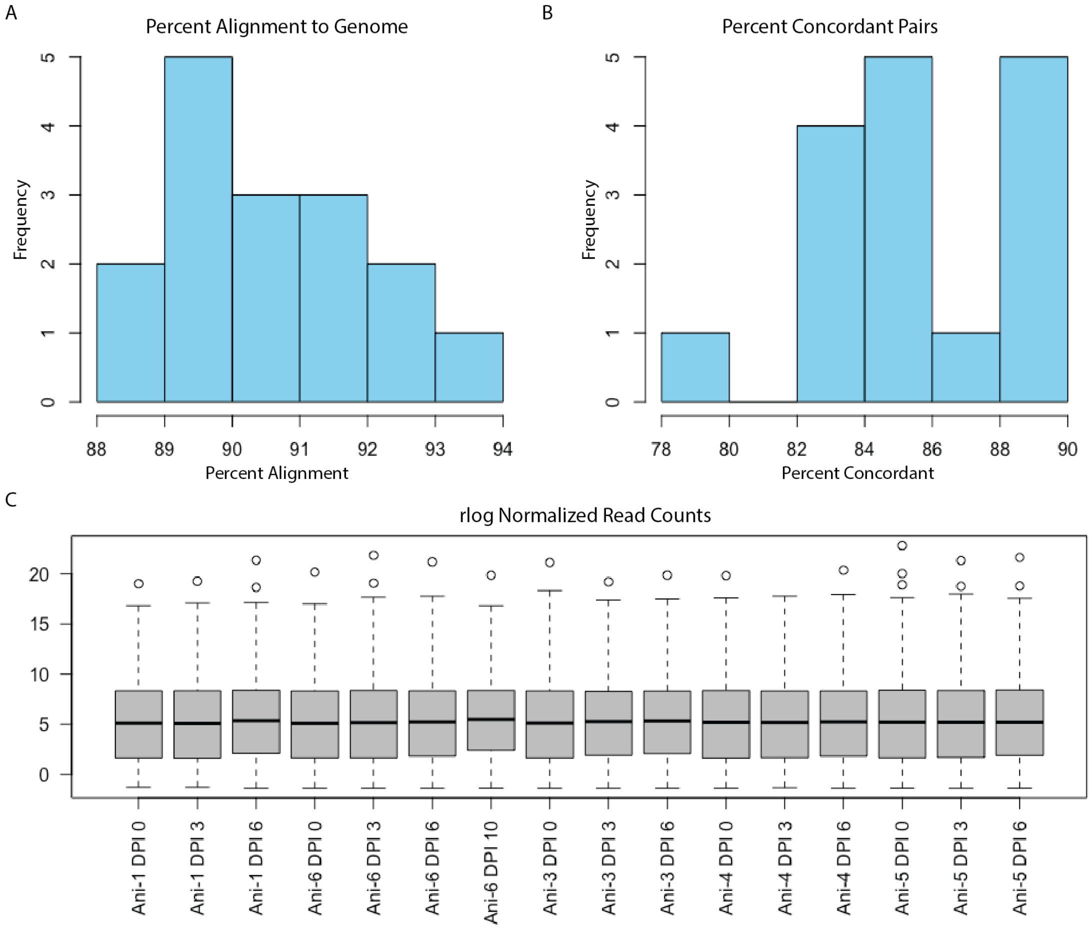

Supplemental Figure S1: Overview of the Alignment to Cynomolgus Macaque Genome. (A) Histogram showing the percentage of reads that aligned to the genome. (B) Percentage of paired reads that aligned concordantly. (C) Box plot of the normalized counts for the data showing that the normalization produced similar distributions.

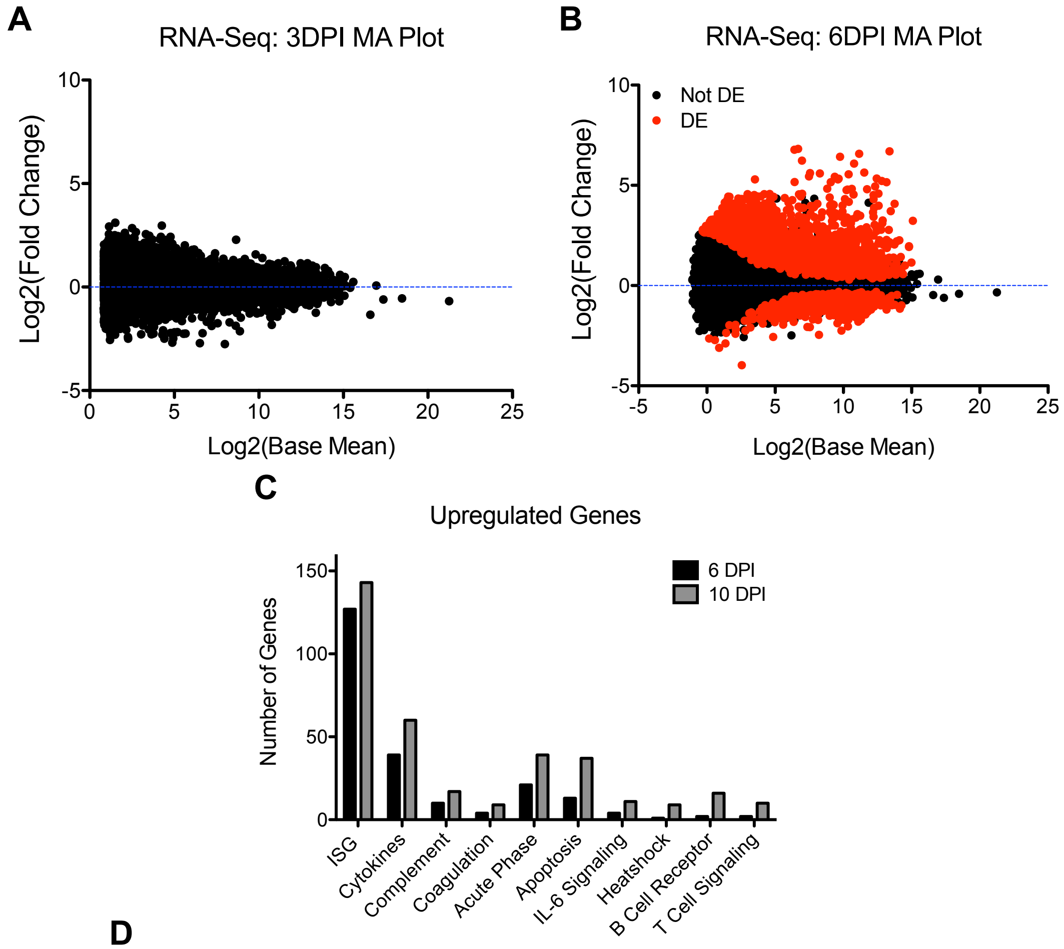

D

	NHP		Human	
Survival	Fatal	Fatal	Fatal	Survivor
EBOV Variant	Makona	Kikwit	Makona	Makona
Route	Mucosal/IM	IM	Various*	Various*
Dose	10, 100, 1000 PFU	1000 PFU	Various*	Various*
Sample Type	Whole Blood	PBMC	Whole Blood	Whole Blood
Interferon Signaling	Upregulated	Upregulated	Upregulated	Upregulated
Cytokines	Upregulated	Upregulated	Upregulated	Upregulated
Acute Phase Response	Upregulated	No Significant Change	Upregulated	Upregulated
T Cell Signaling	No Significant Change	N/A	No Significant Change	Upregulated
Neutrophils	Upregulated	Upregulated	No Significant Change	No Significant Change

Upregulated: the group of genes shows a general trend of increased expression
No Significant Change: the group of genes does not show any significant change in expression
N/A: not explicitly shown, *Route or Dose is unknown

Supplemental Figure S2: Transcriptional Response to EBOV/Mak. Overview of the general transcriptional changes observed in EBOV/Mak infection. **(A)** is an MA-plot of the RNA-Seq data at day 3 post infection. The x-axis represents the log2(base mean) of the counts and the y-axis is the log2(fold change) compared to day 0. Each point is a single gene. Black dots indicate that

the gene is not differentially expressed (Not DE) and red dots indicate differentially expressed genes (DE). **(B)** is a similar plot for day 6 post infection. **(C)** Gene set enrichment for differentially-expressed genes using annotated gene lists (x axis). The y-axis is the number of genes that are up-regulated in that group at day 6 post infection (black) or day 10 post infection (grey). **(D)** is a table outlining the major findings from various EBOV infections. The Kikwit data is from [1,2] and the human data is from [3]. The first 6 rows outline the datasets to show organism (NHP vs. Human), exposure route, EBOV variant (Makona or Kikwit), infectious dose, and sample type. Following this, different gene groups are listed in the left column with their general trend of regulation in the rows across the different groups. Up regulated signifies that the group saw a general trend of up regulation compared to controls. No significant change means there was no strong change in expression compared to controls for that set of genes.

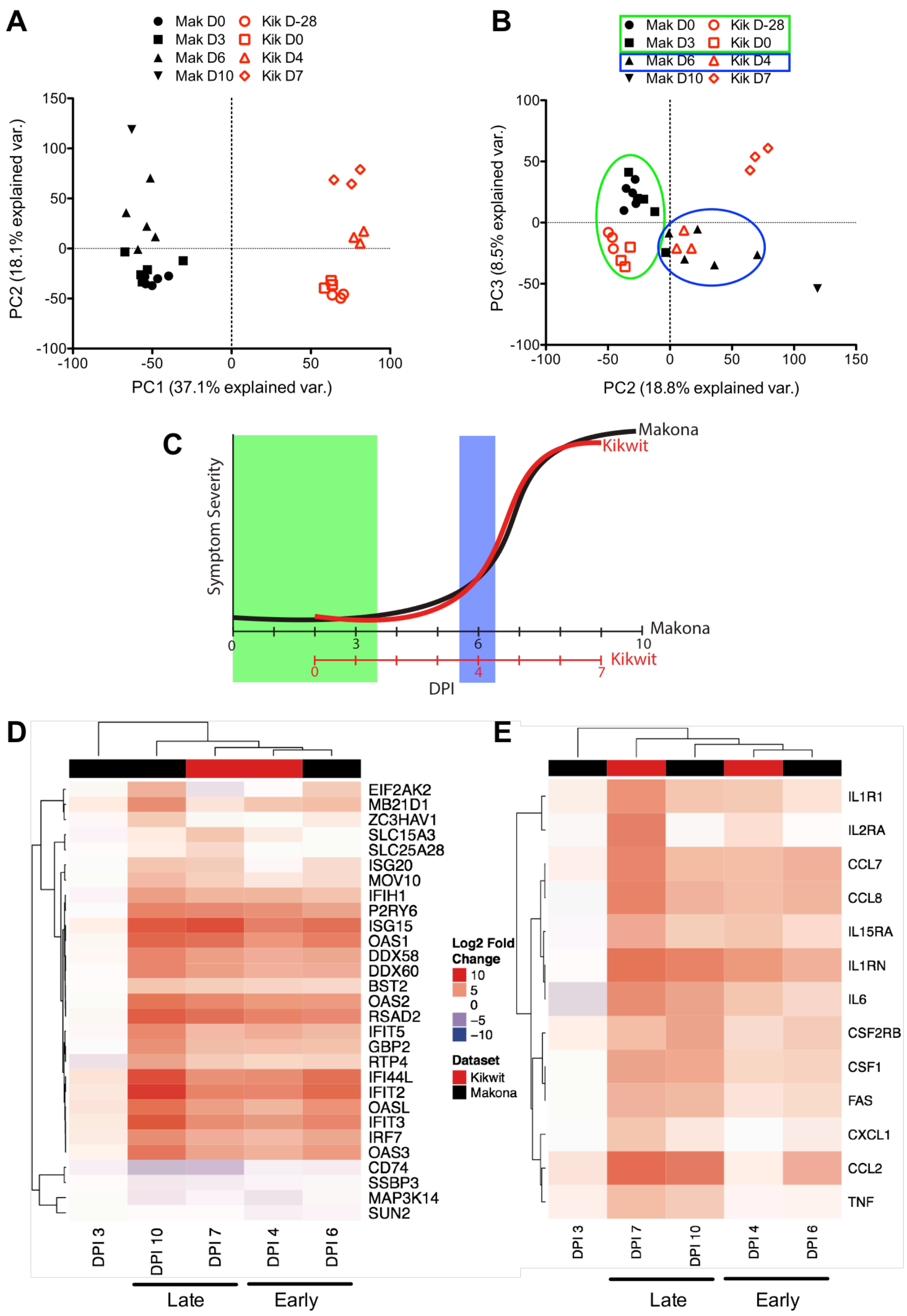

Supplemental Figure S3: Comparison of EBOV/Mak to EBOV/Kik infection. Overview of the comparison of whole blood EBOV/Mak infection to PBMC EBOV/Kik infection. **(A)** is a PCA plot of the gene expression changes with the first principal component on the x-axis and the second principal component on

the y-axis. Black points are from EBOV/Mak infections and red points are from EBOV/Kik infection. The different shapes represent different days as indicated in the legend. **(B)** is a similar plot for the second principal component (x-axis) and the third principal component (y-axis). The green circle is highlighting the pre-infection cluster and relates to the green box in the legend. The blue circle is day 4 and day 6 post infection for EBOV/Kik and EBOV/Mak respectively and related to the blue box in the legend. **(C)** is a generalized graph of the relative disease severity (y-axis) where the higher along the y-axis the more sever the disease course. The x-axis (black) is the days post infection for EBOV/Mak infections and the x-axis (red) is the days post infection for EBOV/Kik infections. The red line (EBOV/Kik) and black line (EBOV/Mak) show the disease progression in the two infections. The green and blue shaded areas represent the clustering seen in **(B)**. **(D)** is a heatmap of many interferon stimulated genes. The rows are genes and the columns are different days post infection from EBOV/Kik (red) or EBOV/Mak (black). **(E)** is a similar heatmap for cytokine genes.

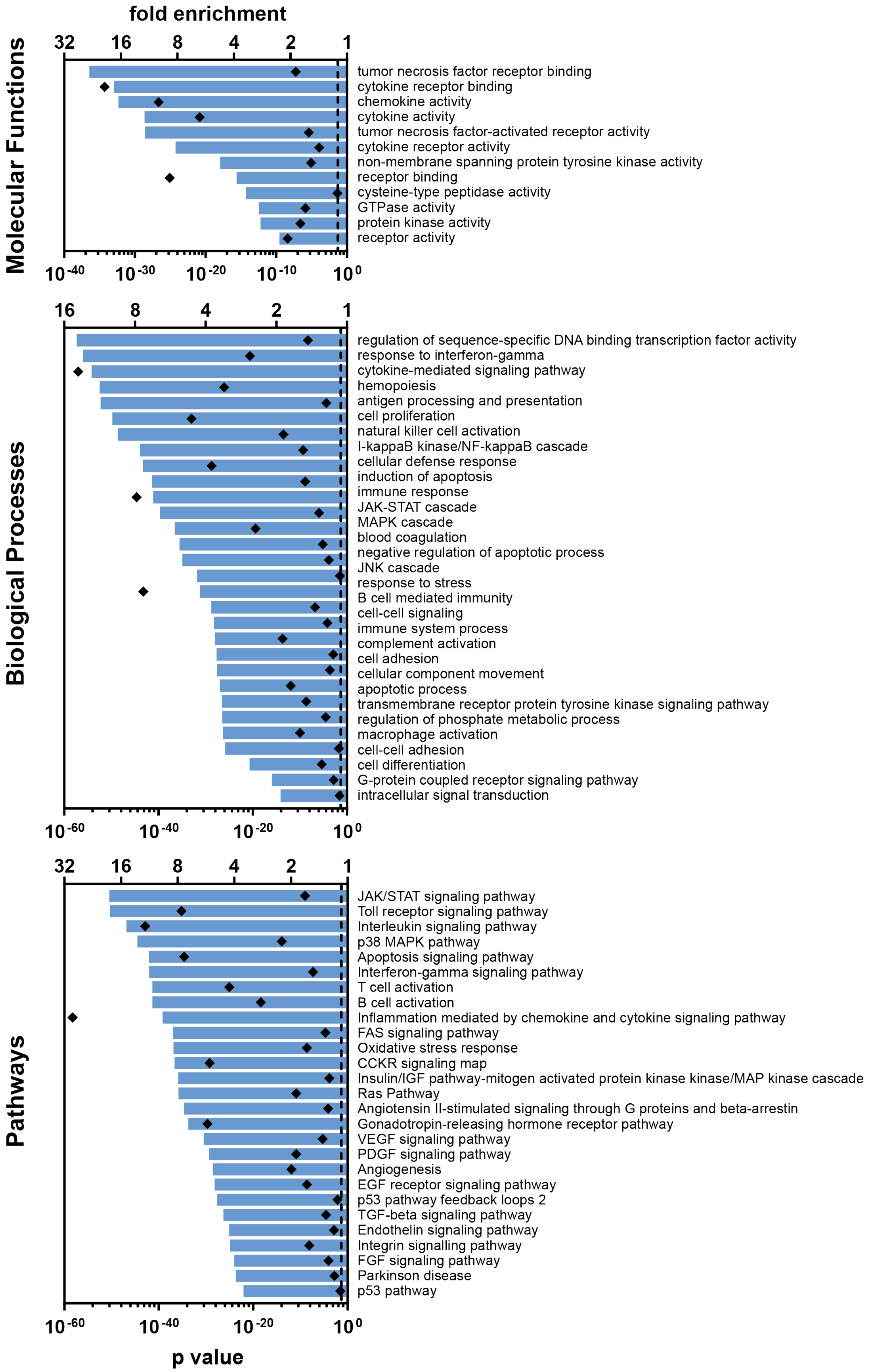
fold enrichment
32
16
8
4
2
1
Molecular Functions
tumor necrosis factor receptor binding
cytokine receptor binding
chemokine activity
cytokine activity
tumor necrosis factor-activated receptor activity
cytokine receptor activity
non-membrane spanning protein tyrosine kinase activity
receptor binding
cysteine-type peptidase activity
GTPase activity
protein kinase activity
receptor activity
10^-40
10^-30
10^-20
10^-10
10^0
16
8
4
2
1
Biological Processes
regulation of sequence-specific DNA binding transcription factor activity
response to interferon-gamma
cytokine-mediated signaling pathway
hemopoiesis
antigen processing and presentation
cell proliferation
natural killer cell activation
I-kappaB kinase/NF-kappaB cascade
cellular defense response
induction of apoptosis
immune response
JAK-STAT cascade
MAPK cascade
blood coagulation
negative regulation of apoptotic process
JNK cascade
response to stress
B cell mediated immunity
cell-cell signaling
immune system process
complement activation
cell adhesion
cellular component movement
apoptotic process
transmembrane receptor protein tyrosine kinase signaling pathway
regulation of phosphate metabolic process
macrophage activation
cell-cell adhesion
cell differentiation
G-protein coupled receptor signaling pathway
intracellular signal transduction
10^-60
10^-40
10^-20
10^0
32
16
8
4
2
1
Pathways
JAK/STAT signaling pathway
Toll receptor signaling pathway
Interleukin signaling pathway
p38 MAPK pathway
Apoptosis signaling pathway
Interferon-gamma signaling pathway
T cell activation
B cell activation
Inflammation mediated by chemokine and cytokine signaling pathway
FAS signaling pathway
Oxidative stress response
CCKR signaling map
Insulin/IGF pathway-mitogen activated protein kinase kinase/MAP kinase cascade
Ras Pathway
Angiotensin II-stimulated signaling through G proteins and beta-arrestin
Gonadotropin-releasing hormone receptor pathway
VEGF signaling pathway
PDGF signaling pathway
Angiogenesis
EGF receptor signaling pathway
p53 pathway feedback loops 2
TGF-beta signaling pathway
Endothelin signaling pathway
Integrin signalling pathway
FGF signaling pathway
Parkinson disease
p53 pathway
10^-60
10^-40
10^-20
10^0
p value

Supplemental Figure S4: Gene ontology and pathway analysis of NanoString non-human primate gene expression codeset. The 769 gene identifiers were submitted to the PANTHER Classification System (version 11.1) for gene list analysis. Overrepresented molecular functions, biological processes, and pathways based on this list relative to all human genes in the database are shown. These fold enrichment factors are shown as bars, while the associated p values derived from Mann-Whitney rank-sum tests are shown as overlaid black diamonds. Only ontology terms or pathways with $p < 0.05$ are shown. All significant terms for the molecular functions and pathways are shown, while some redundant terms have been removed the list of biological processes.

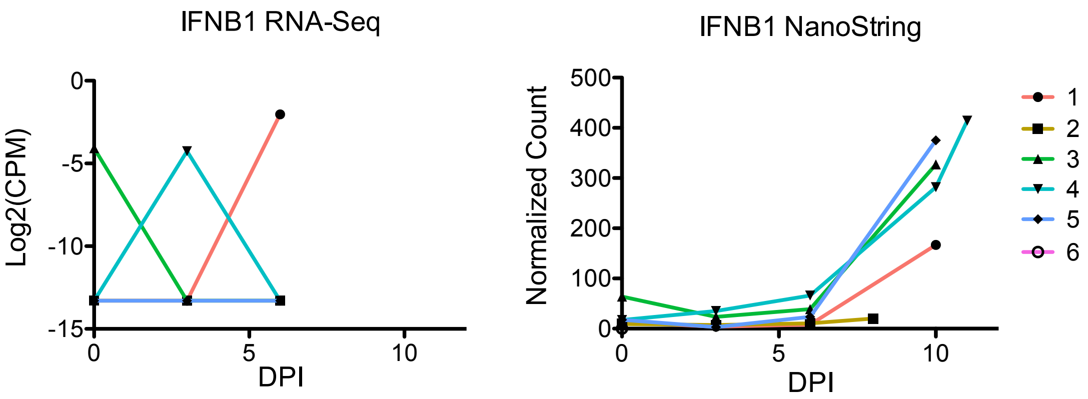

Supplemental Figure S5: Upregulation of interferon beta in NHPs can only be detected using NanoString. (A) IFNB1 expression in RNA-Seq data shown as log2(CPM) over day post-infection (DPI). No animal had any significant counts (sum counts across time for individual animal < 4). **(B)** IFNB1 expression in NanoString as normalized counts over DPI. Background for this platform was defined as 16 counts as determined by negative control lanes.

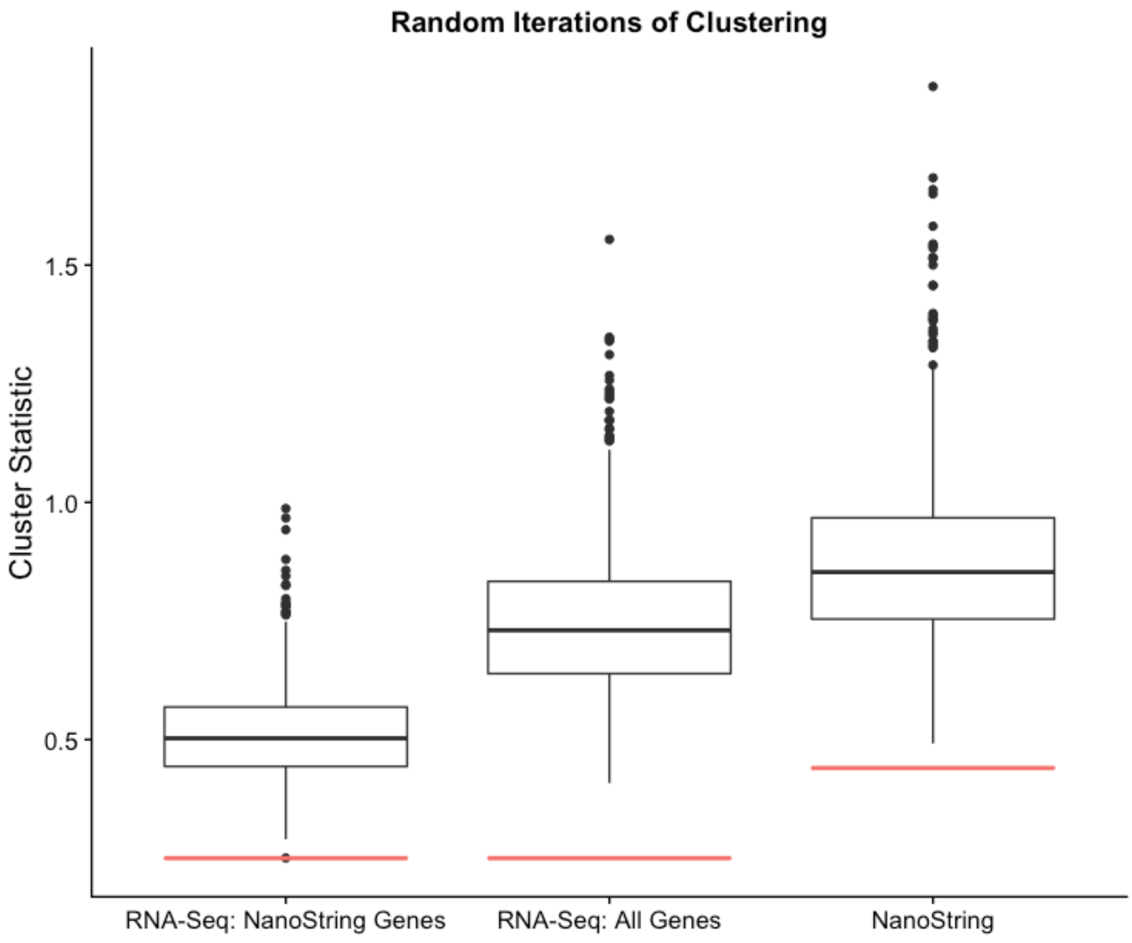

Supplemental Figure S6: Comparison of the ability of 1000 iterations of randomly selected genes to cluster (box plot) compared to the 41 gene set (red bar). A smaller the cluster statistic indicates better clustering. In all cases, the red bar is below all the randomly iterated gene sets.

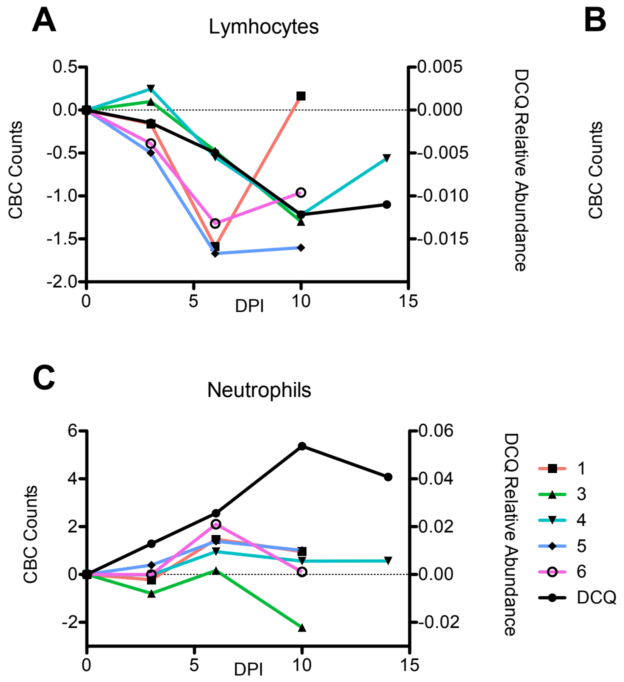

Supplemental Figure S7: Comparison of CBC data to DCQ predictions.
(A) Comparison of the CBC data to the predicted relative abundance using DCQ for neutrophil amount. Day post-infection (DPI) is shown on the x-axis. The secondary (right) y-axis shows the DCQ predicted relative abundance (black line). This corresponds to the black line. The $\log_2$(fold) of the CBC data relative to day 0 for each individual animal is seen on the primary (left) y-axis (colored lines). This corresponds to the colored lines. Each animal has a different line pattern to distinguish them.

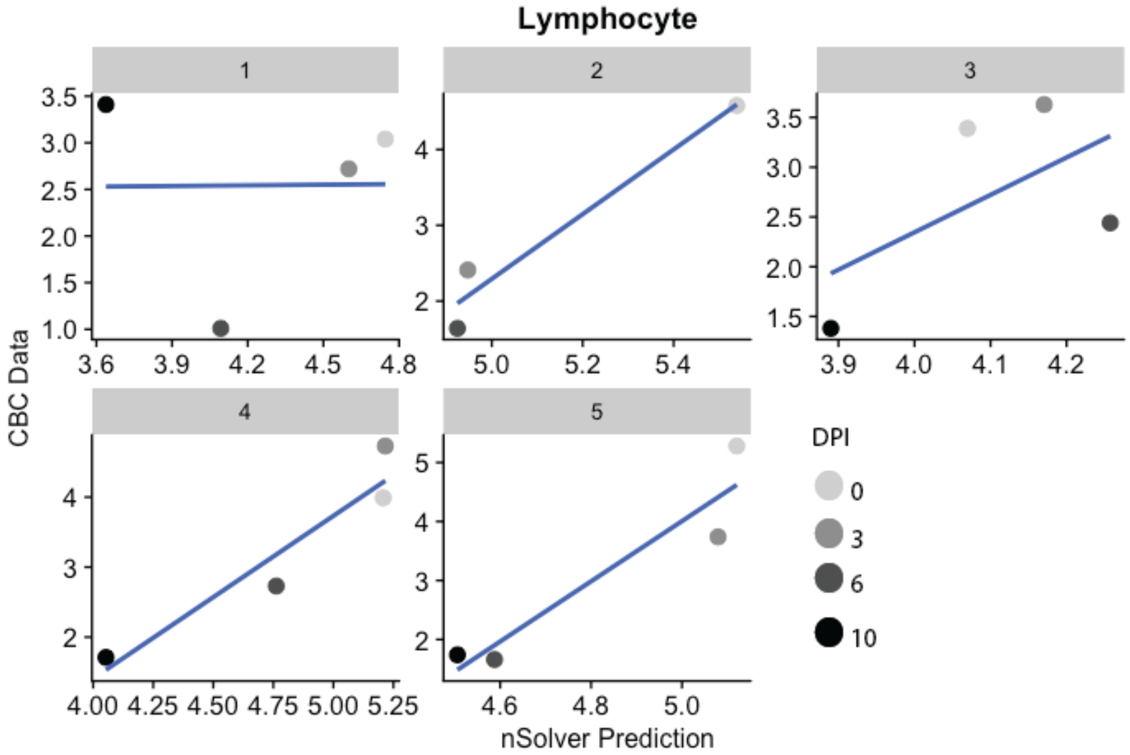

Supplemental Figure S8: nSolver predictions compared to CBC data for individual animals. The x-axis shows the predicted changes in lymphocytes populations as predicted by the nSolver software for different animals. The points represent different DPI. The y-axis is the CBC data for changes in lymphocytes. The blue line represents the linear regression line to fit the data.

Supplemental References

1. Caballero, I. S. *et al.* In vivo Ebola virus infection leads to a strong innate response in circulating immune cells. *BMC Genomics* **17,** (2016).
2. Cilloniz, C. *et al.* Functional genomics reveals the induction of inflammatory response and metalloproteinase gene expression during lethal Ebola virus infection. *J. Virol.* **85,** 9060–8 (2011).
3. Liu, X. *et al.* Transcriptomic signatures differentiate survival from fatal outcomes in humans infected with Ebola virus. *Genome Biol.* **18,** 4 (2017).
